# MONALISA for stochastic simulations of Petri net models of biochemical systems

**DOI:** 10.1186/s12859-015-0596-y

**Published:** 2015-07-10

**Authors:** Pavel Balazki, Klaus Lindauer, Jens Einloft, Jörg Ackermann, Ina Koch

**Affiliations:** 1 Department of Molecular Bioinformatics, Institute of Computer Science, Cluster of Excellence “Macromolecular Complexes”, Johann Wolfgang Goethe-University Frankfurt am Main, Robert-Mayer-Straße 11-15, Frankfurt am Main, 60325 Germany; 2 Sanofi Aventis Deutschland GmbH, Industriepark Höchst H831, Frankfurt am Main, 65926 Germany

**Keywords:** Stochastic simulation algorithm, Gillespie’s algorithm, Petri net, MonaLisa, Insulin receptor recycling

## Abstract

**Background:**

The concept of Petri nets (PN) is widely used in systems biology and allows modeling of complex biochemical systems like metabolic systems, signal transduction pathways, and gene expression networks. In particular, PN allows the topological analysis based on structural properties, which is important and useful when quantitative (kinetic) data are incomplete or unknown. Knowing the kinetic parameters, the simulation of time evolution of such models can help to study the dynamic behavior of the underlying system. If the number of involved entities (molecules) is low, a stochastic simulation should be preferred against the classical deterministic approach of solving ordinary differential equations. The Stochastic Simulation Algorithm (SSA) is a common method for such simulations. The combination of the qualitative and semi-quantitative PN modeling and stochastic analysis techniques provides a valuable approach in the field of systems biology.

**Results:**

Here, we describe the implementation of stochastic analysis in a PN environment. We extended MonaLisa - an open-source software for creation, visualization and analysis of PN - by several stochastic simulation methods. The simulation module offers four simulation modes, among them the stochastic mode with constant firing rates and Gillespie’s algorithm as exact and approximate versions. The simulator is operated by a user-friendly graphical interface and accepts input data such as concentrations and reaction rate constants that are common parameters in the biological context. The key features of the simulation module are visualization of simulation, interactive plotting, export of results into a text file, mathematical expressions for describing simulation parameters, and up to 500 parallel simulations of the same parameter sets. To illustrate the method we discuss a model for insulin receptor recycling as case study.

**Conclusions:**

We present a software that combines the modeling power of Petri nets with stochastic simulation of dynamic processes in a user-friendly environment supported by an intuitive graphical interface. The program offers a valuable alternative to modeling, using ordinary differential equations, especially when simulating single-cell experiments with low molecule counts. The ability to use mathematical expressions provides an additional flexibility in describing the simulation parameters. The open-source distribution allows further extensions by third-party developers. The software is cross-platform and is licensed under the Artistic License 2.0.

**Electronic supplementary material:**

The online version of this article (doi:10.1186/s12859-015-0596-y) contains supplementary material, which is available to authorized users.

## Background

Chemical and thus biochemical systems, can be modeled at different levels of abstraction. The choice and level of the method depends on the available data. With the development of the *omics*-technologies we get many different types of experimental data, ranging from NGS (Next Generation Sequencing) data to image data. It is indispensable to check the quality and completeness of the data to decide which method should be applied for theoretical modeling. In this context, it is essential to provide mathematical formalisms which can handle and combine different levels of abstraction. This can then lead to assurance of several sound analysis techniques and an intuitive graphical interface. Here, we use Petri nets (see next subsection) — a formalism that exhibits all these properties. State-of-the-art tools implementing hybrid Petri nets include commercial tools, e.g., *Cell Illustrator* [1,2], and colored Petri nets, e.g., *CPN* [3]. Our aim is to provide an open-source, easily extendable tool for biochemical Petri nets that allow to model the classical P/T networks as well as the Gillespie’s stochastic simulation approach.

If there exists kinetic data in a sufficient amount we can start a quantitative modeling. Mathematical models of (bio)chemical reaction systems are usually formulated in terms of ordinary differential equations (ODE). The simulation of such models is performed numerically, and the result is strictly deterministic. This approach ignores stochastic fluctuations which are important for biological systems operating with low molecule numbers. The concept of Chemical Master Equations (CME) aims to describe stochastic fluctuation in reaction systems. For a more detailed introduction we refer to the textbook by Atkins [4].

The CME is a countable, but infinite, system of first-order differential equations that determines time evolution of the *probabilities* of the discrete states of a system. A numerical solution of this equation is infeasible. Alternatively, the Monte Carlo simulation method can be applied to compute valid trajectories through the state space of the system. For this purpose, Gillespie [5] proposed an efficient stochastic simulation algorithm (SSA). For the initial state of a chemical system, the Gillespie’s method simulates the evolution of the number of molecules by estimating when and which reaction would be occurring next. Several implementations of the Gillespie’s method have already been proposed to improve the computational performance. The *approximate* SSA [6] facilitates a significant speed-up factor.

### Petri nets

Petri net (PN) is a powerful mathematical concept [7], which is widely applied for modeling systems of chemical reaction, metabolic pathways [8], signaling pathways [9], or gene expression networks [10]. The main idea is the consequent distinction between passive and active parts of the network, enabling for a sound treatment of concurrency. Here, we will give briefly the main definitions which are essential for understanding the idea described in our approach.

#### **Definition1** (Petri net).

A Petri net (PN) is a directed bipartite graph PN = (P, T, E, f(e), *m*(*P*_*i*_)) with

- P and T are disjoint sets, *P*∪*T* is the set of all vertices. P is the set of *places*, T is the set of *transitions*,
- E ⊆((*P*×*T*)∪(*T*×*P*)) is the set of directed edges,
- $f(e): E \rightarrow \mathbb {N}_{+} $ is the *weight function* which assigns a positive integer weight to each edge *e*∈*E* and
- $m(P_{i}): P \rightarrow \mathbb {N}_{0}$ is the *marking* which assigns a positive integer number of tokens to each place *P*_*i*_∈*P*.

Places (states or species) represent passive and transitions (reactions) active elements. Movable objects are called *tokens* and are located on the places. A token distribution (*marking*) represents the number of entities of the corresponding species and defines a system state. The dynamics are modeled by the movements of tokens through *firing* of transitions, applying firing rules.

#### **Definition2**.

(Pre- and post-places, firing of a transition)

- ∙*T*_*i*_:={*P*_*j*_∈*P* | (*P*_*j*_, *T*_*i*_)∈*E*} is the set of the *pre-places* of transition *T*_*i*_, i.e., the set of all places which have an outgoing edge to *T*_*i*_.
- *T*_*i*_∙:={*P*_*j*_∈*P* | (*T*_*i*_, *P*_*j*_)∈*E*} is the set of the *post-places* of transition *T*_*i*_, i.e., the set of all places which have an incoming edge from *T*_*i*_.
- Transition *T*_*i*_ is *active* and can *fire* iff ∀*P*_*j*_ ∈∙*T*_*i*_:*f*(*P*_*j*_,*T*_*i*_)≤*m*(*P*_*j*_).

Pre- and post-transitions are defined analogously to pre- and post-places.

A transition can only fire, if the number of tokens on all pre-places of the transition is equal to or greater than the weights of the edges between the corresponding places and the transition. In this case, the transition is *active* or *enabled*. Firing of a transition consumes tokens from the pre-places and adds tokens to the post-places, according to the weights of the corresponding edges. We extend the standard definition of PN by introducing *constant places*. The number of tokens on a constant place is not affected by the firing of transitions but is determined by a user-defined mathematical expression. For instance, pulsatile secretion of insulin in a model of insulin signaling can be described as

$$(1400 \ cos(Time / 300 \ 2\pi) + 1600) $$

where the variable *Time* stands for the simulated time (in seconds).

In the context of (bio)chemical systems, places represent chemical compounds (e.g. insulin, insulin receptor), complexes (e.g. receptor-ligand complex) or different states (e.g. inactive or active receptor), and transitions describe chemical reactions. The number of tokens on a place represents the number of molecules of the corresponding biochemical species. In signaling or gene expression PN, the number of tokens can describe the activation state (on/off) or the strength of a response. An overview of the PN formalism and its application to biology has already been earlier described [11,12].

### Available software

MONALISA [13] is an open-source software for creation, visualization and analysis of PN. MONALISA implements several analysis techniques such as invariant analysis (implemented in C, [14]), topology properties, knockout analysis, and other, supporting a broad range of file formats like PNML, PNT, SPEED, SBML (read: all levels and versions of SBML Core, write: SBML core Level 2, Version 4), KGML and DAT. MONALISA provides the possibility to easily extend its functionality by new modules. It also supports the annotation with MIRIAM identifiers and SBO terms. An interface to SED-ML [15] is planned.

Here, we introduce a module which extends the functionality to applications of dynamic simulations. The applied firing rules determine the strategy to choose the next transition to fire and the time point at which the event takes place. Stochastic firing rules simulate the dynamics of the stochastic kinetic of a mass action reaction system inside a cell. Alternative tools for the stochastic analysis of biochemical PN are Snoopy [16] and VANTED [17]. VANTED is an open-source solution but without any simulation module. Snoopy is not open-source and aims at providing the entire types of PN mainly for the Petri net community. Modeling for biology is one aspect among many others. MonaLisa is focused on biological applications and covers another spectrum than Snoopy, ranging from general network analysis to specific decomposition methods at steady-state as well as non-steady-state conditions. Moreover, MONALISA includes a simulation mode with multiple parallel simulations. For a more detailed comparison of MONALISA and Snoopy see Table 1. The table compares the different features of both tools, like existing simulation modes, analysis techniques, availability, and supported file formats.

**Table 1:** **A comparison of** MONALISA **and Snoopy**

	**Snoopy**	**MonaLisa**
Availability	closed source	open source
Biological terminology	no	yes
Annotation facilities	no	MIRIAM Identifier and SBO terms
Supported analysis techniques^1^	no	P-Invariants, T-Invariants, Maximal Common Transitions sets, distance matrix, T-Cluster, Knock-out, Minimal Cut sets, node degrees
Color highlighting of analysis results	no	yes - for Invariants, MCT-sets, and Knock-outs
Supported simulation modes	P/T-net animation, Gillespie, FAU	Asynchronous, Synchronous, Stochastic, (Gillespie)
Supported Petri net classes	19 - for example: P/T-net, Fault Tree, Extended Fault Tree, Freestyle Net	P/T-nets
Supported input file formats	ANDL, CANDL, APNN, SBML, PED, PNML, TINA, CSV, DNF	APNN, KEGG, METATOOL, PNML, PNT, SBML, SPEED
Supported output file formats	19 - for example: ANDL, CANDL, Maria, PEP, Prod	APNN, METATOOL, PNML, SBML, SVG, PNG, TXT, PNT
Supported Operation Systems	Windows, MacOS X, Linux (selected distributions)	Windows, MacOS, Linux
Software Platform	C++	Java
Editor UI	yes	yes

^1^Snoopy does not involve analysis techniques. These have to be approached via different file formats for other tools.

StochPy [18] is another tool for stochastic simulation of biological processes, but is not PN-based. It uses the PySCeS [19] model description language — a text-based model description technique. It provides an integrated statistical output analysis (auto-correlations, propensities, moments, waiting times) and can be easily extended due to its integration with other Python libraries. Implementations of the exact and the approximate SSA are offered, but StochPy provides neither an intuitive graphical user interface nor any PN and topological analysis method.

## Implementation

MONALISA is an open-source software (see Additional file 1) distributed under the Artistic License 2.0. It is accessible to modifications and allows adjustment and extension of its functionality by experienced users. A description of all features of the simulation module is given in the documentation file MonaLisa_Documentation.pdf (Additional file 2) provided in the supplementary.

### Libraries

The simulation module is implemented in Java, version 1.7, as a plug-in of MONALISA. The plotting functionality exploits the JFreeChart-library [20]. The integration of the library exp4j [21] develops the option to evaluate user-defined mathematical expressions. The Java class *HighQualityRandom* [22] implements the standard pseudo random number generator (RNG) *Ran* defined by Press *et al.* [23]. This RNG is a good compromise between speed and cryptographic randomness and appropriate for all algorithms described here. For a more detailed discussion of the choice of RNGs for simulations we refer to the textbook of Knuth [24] and the numerous literature published on this topic, see, e.g., [25-29].

### Modes

PN modeling addresses a broad range of scenarios in systems biology with various goals and requirements. To account for the different needs, we implemented four simulation modes in MONALISA, ranging from simple synchronous and asynchronous modes to the well-established Gillespie’s algorithm for stochastic simulation:

1. **Asynchronous:** One randomly chosen transition fires per simulation step, without any time consumption. All active transitions have the same probability to fire.
2. **Synchronous:** Multiple active transitions fire simultaneously per step. By default, the simulator tries to fire all active transitions at once. Transitions which share pre-places are called *concurrent* and compete for the tokens of the shared pre-places. If the number of tokens on such a place is not sufficient for all enabled post-transitions, transitions to fire are randomly chosen with equal probability until all tokens are consumed. The simulator also allows to select the fraction of active transitions to fire. If the fraction is less than 100%, transitions are randomly selected with equal probability in each step.
3. **Stochastic:** An enabled transition *T*_*i*_ has to wait a defined time *dt* before it can fire. The waiting time *dt* is simulated for each transition as an exponential distributed random variable *E* *x* *p*(*λ*), a detailed description is available in the textbook of D.J Wilkinson [30]. The parameter *λ* is the firing rate specified by the modeler for the particular transition. Among all enabled transitions only the transition with the lowest waiting time fires. If several transitions have the lowest waiting time, one of them is selected randomly. To speed up the simulation, waiting times are recomputed only for transitions with changed number of tokens on the pre-places. Waiting times of the post-transitions of constant places are recomputed in every step.
4. **Stochastic simulation algorithm:** This algorithm implements the exact and approximate SSA. The exact SSA implements the direct method of Gillespie [5,30], applying an internal data structure similar to the “dependency graph” introduced by Gibson and Bruck [31] for better performance.
  The exact SSA consists of four steps:
  i Computing the rates of all reactions based on the stochastic rate constants *c*(*T*_*i*_) and the number of reactant molecules. We refer to the textbook of Wilkinson [30] for a detailed description.
  ii The next firing time *dt* is computed by
    $$dt = -\frac{ln(1-U_{1})}{\text{sum of reaction rates}} $$
    from a uniformly distributed (in the interval [0,1]) random number *U*_1_.
  iii A reaction *i* is chosen such that the following equation is satisfied
    $$\sum_{j=1}^{i-1}r(T_{j}) < U_{2} \ \text{sum of all rates} \leq \sum_{j=1}^{i}r(T_{j}) $$
    for a second uniformly distributed random number *U*_2_.
  iv The number of molecules is updated according to the chosen reaction *i* and the time is increased by *dt*.
  The approximate SSA integrates the *τ*-leaping algorithm of Gillespie and Petzold [32,33]. In brief, the algorithm chooses a time interval *τ* and decides how many times each reaction will occur in this period. The time interval should be small enough so that the reaction rates do not change significantly during this interval. To avoid negative populations, reactions that do not have sufficient reactants to fire at least 20 times are considered as *critical* and are simulated in an exact way only.
  At the beginning of each simulation step critical reactions are determined. The algorithm computes two firing times – one for the *non-critical reactions* *τ*_1_ according to [33] and a second time *τ*_2_ for the *critical reactions*.
  If *τ*_1_<*τ*_2_, no critical reaction occurs. For each non-critical reaction, the number of its occurrences is generated as a random Poisson variable with the mean value *r*(*T*_*i*_) *τ*_1_, and the reaction is executed according to the chosen number of times. If *τ*_2_<*τ*_1_, one critical reaction is chosen like in the exact SSA and the number of occurrences of the non-critical reactions are chosen as for the first case.

### Converting input data

In contrast to ODE-based approaches, the stochastic methods operate with numbers of molecules instead of concentrations. Therefore, the continuous biological parameters like concentrations of chemical compounds and the reaction rate constants must be converted to molecule-based data. So, the user has to provide the volume of the simulated environment as an additional parameter, and a preprocessor converts concentrations and mass action reaction rate constants to the appropriate numbers of molecules and stochastic rate constants, respectively:

$$ n_{X} = N_{A} \ [\!X] \ V $$

where *n*_*X*_ is the number of molecules of compound *X*, [*X*] the concentration of compound *X*, *N*_*A*_=6·10^23^ the Avogadro constant and *V* the reaction volume.

The conversion of the *mass action* rate constant *k* to the *stochastic* rate constant *c* depends on the order of the reaction. The mass action reaction rate constant is given in units of *M* *s* ^−1^ for a zero order reaction, in units of *s* ^−1^ for a first order reaction, in units of *M* ^−1^ *s* ^−1^ for a second order reaction, and in general, in units of *M* ^−(*x*−1)^ *s* ^−1^ for a reaction of the order of *x*. The mass action reaction constant *k* gives the reaction rate for standardized concentrations of 1 M (i.e., *N*_*A*_ molecules in 1 l reaction volume). To get the reaction rate constant for the number of molecules we have to divide *k* by (*V* *N*_*A*_)^(*x*−1)^ and get

$$c \sim k / (V \ N_{A})^{x-1}. $$

A substance (pre-place) *P*_*j*_ may contribute with high stoichiometric order (i.e., weight *f*(*P*_*j*_,*T*_*i*_)) to the reaction *T*_*i*_. The combinatorial factor

$$\prod\limits_{P_{j} \in \bullet T_{i}} f(P_{j},T_{i})! $$

accounts for the stoichiometric factors of the species *P*∈∙*T*_*i*_. Finally, we get the general formula

$$c(T_{i})= \frac{k(T_{i}) \ \prod_{P_{j} \in \bullet T_{i}} f(P_{j},T_{i})!}{(V \ N_{A})^{(x-1)}}. $$

This equation can be simplified to:

- *c*=*k* *V* *N*_*A*_ for zero-order reaction,
- *c*=*k* for first-order reaction,
- $c = \frac {k}{V \ N_{A}}$ for second-order reaction of the form *A*+*B*→*C*, and
- $c = \frac {2 k}{V \ N_{A}}$ for second-order reaction of the form 2*A*→*B*.

### Mathematical expressions

Reaction rate constants and the number of tokens on constant places can be defined by mathematical expressions which may contain names of non-constant places and the simulated time as variables.

#### Supported syntax

The evaluation of a mathematical expression is based on the free Java library exp4j [21]. It supports numerical input in standard and scientific notations. Supported operators and functions are listed in Table 2.

**Table 2:** **Operators and functions which are supported by mathematical expressions for describing the number of tokens (or concentrations) on constant places or reaction rate constants**

**Operators**	
Addition	2+2
Subtraction	2−2
Multiplication	2·2
Division	2/2
Exponentiation	2 ˆ 2
Sign operators	+2−(−2)
Modulo	2 % 2
**Functions (use as “func(x)”)**	
abs	absolute value
acos	arc cosine
asin	arc sine
atan	arc tangent
cbrt	cubic root
ceil	nearest upper integer
cos	cosine
cosh	hyperbolic cosine
exp	Euler’s number raised to the power (eˆx)
floor	nearest lower integer
log	natural logarithm (base e)
sin	sine
sinh	hyperbolic sine
sqrt	square root
tan	tangent
tanh	hyperbolic tangent
div(x,y)	integer division, e.g., div(28,24) returns 1

#### Conditional expression

It is possible to use multiple conditional expressions to define the reaction rate constant or the number of tokens on a constant place. The syntax has the form



Each condition is composed of the three parts:



with mathematical expressions [expression 1] and [expression 2]. The operator ([operator]) is one of “ =”, “ <”, “ >”, “ <=”, or “ >=”, describing comparisons. Multiple conditional expressions can be separated by a semicolon. The value of the first conditional expression whose conditions are satisfied is returned. If none of the described cases can be satisfied, 0 is returned. For example, the number of insulin molecules should be 1000 for the first 5 minutes, 100 for the next 5 minutes and 0 afterwards:



As long as the reaction time is below 300, the first conditional expression is valid, and the value 1000 is returned. If the reaction time exceeds 5·60, the second conditional expression becomes valid, and the number of insulin molecules is set to 100. After the reaction time of 10·60 seconds the number of insulin molecules drops down to zero.

Mathematical expressions are evaluated after each simulation step. In combination with time-dependent expressions the Gillespie algorithm may lead to discretization artifacts in the case of the combination of a very large waiting time with a fast variation of the conditional expressions on a similar time scale. The discretization artifacts are negligible for standard applications. In further versions of MONALISA, we will implement the rejection method [34] to provide a more robust algorithm for this non–homogeneous Poisson process.

## Results and discussion

**Figure 1:**
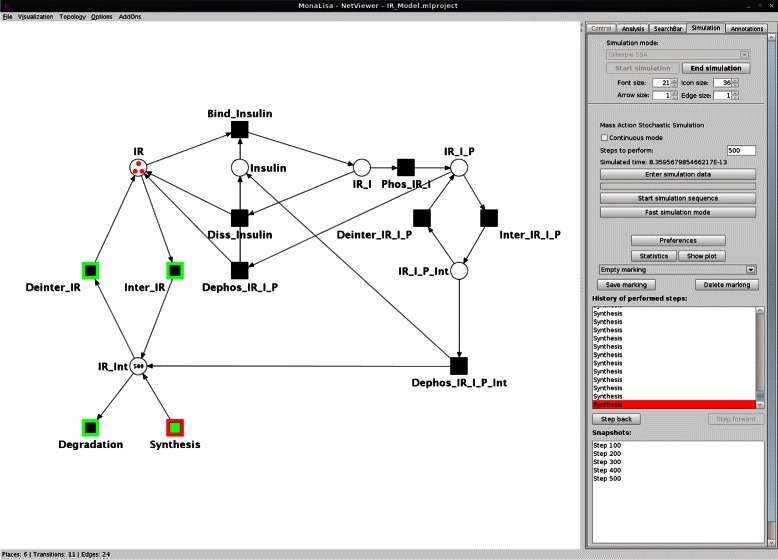
Graphical User Interface (GUI) of the simulation module. The GUI of MONALISA allows to control and to keep track of the simulation. The left part depicts the graphical representation of the PN model. The number of tokens is written on the places and transitions are colored according to their state (active/inactive, last fired). The right part shows the controls of the simulator module.

MONALISA provides a PN editor, including visualization and a special graphical user interface for the simulation mode (see Figure 1). The simulation process is visualized by showing the current number of tokens on the places, highlighting with different colors active transitions and transitions which fired last. The user may change interactively the number of tokens on places and select manually a transition to fire.

Four different simulation modes can be chosen - asynchronous, synchronous, stochastic and the SSA. Various useful features are available:

- History of fired steps,
- Statistic information (number of fired steps, number of firings each transition has performed),
- Saving and loading of marking,
- Saving and loading of the simulation set–ups, including the marking, firing rates, reaction rate constants, mathematical expressions, and other information in XML format,
- Plot of simulation results,
- Export of simulation results into a tab-stop separated file,
- Application of mathematical expressions to describe rate constants and the number of tokens.

The concept of constant places is an assistant feature of MONALISA. Constant places are ignored in the firing step, though they contribute to the transitions’ activation states. Constant places are useful for modeling boundary conditions, external factors or inhibitory effects.

For biochemical systems, the stochastic and the Gillespie’s algorithm (SSA) are the most interesting simulation modes. Each stochastic simulation of identical initial parameters will have a different outcome. To reproduce a simulation, the user may want to set the seed of the Pseudo Random Number Generator (PRNG). Repetition of simulations with a fixed seed will always produce identical results.

Apart from the exact SSA, the simulator implements the approximate SSA with improved performance for systems with fast reactions or large molecule counts [6]. Up to 500 parallel simulations of identical initial parameters can be performed and take full advantage of multi-core processors.

### Case study of insulin receptor (IR) recycling

We want to explain modeling and simulating of the complex biochemical system of insulin receptor activation and recycling. This case study intends to demonstrate a possible workflow but not a thorough analysis of the IR system.

Insulin is an important hormone that regulates various cellular processes, among others the intake of glucose by such tissues as fat, muscle and brain or glucose production by the liver. The response of a cell to an elevated insulin level is mediated by the insulin receptor, which is mainly located in the cell surface membranes. Impairments in the key components of the IR regulatory system can cause diseases such as the *metabolic syndrome* or *type 2 Diabetes mellitus*.

**Figure 2:**
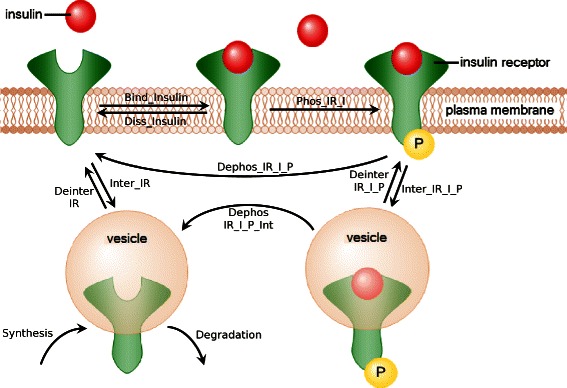
Insulin receptor recycling model. The insulin molecule can bind to a free receptor (*Bind_Insulin*) which leads to the autophosphorylation and activation of the IR (*Phos_IR_I*). The active receptor can be deactivated in the membrane (*Dephos_IR_I_P*) or internalized (*Inter_IR_I_P*) into cytosol where it is deactivated (*Dephos_IR_I_P_Int*). Insulin is degraded in the cytosol, and the free receptor can either be transported back to the membrane (*Deinter_IR*) or be degraded. The internal receptor pool is maintained by the synthesis of new IR.

**Figure 3:**
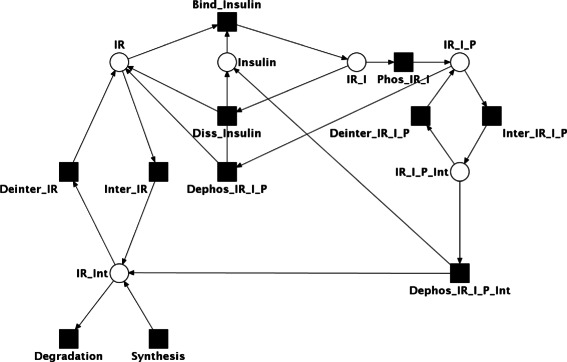
The model of insulin receptor recycling according to Figure 2 is represented as a Petri net. Places are drawn as circles and transitions as black squares.

Figure 2 gives an overview of the processes in the model. Figure 3 depicts the corresponding PN. It consists of 6 places and 11 transitions. The unbound inactive insulin receptor is modeled by the place *IR*, the free insulin by the place *Insulin*. We consider only a single cell. The insulin concentration is regulated by external processes. Consequently, we choose a *constant* place to model the time-dependent external insulin level. Insulin can bind (transition *Bind_Insulin*) to the free receptor. The receptor-ligand complex (place *IR_I*) can dissociate again (transition *Diss_Insulin*). Alternatively to the binding of insulin, the free receptor can be internalized to the cytoplasm (transition *Inter_IR*) and is added to the internal receptor pool (place *IR_Int*).

As soon as insulin is bound to the receptor, the receptor-ligand complex can be phosphorylated and becomes activated (transition *Phos_IR_I*). The bound phosphorylated receptor is represented by the place *IR_I_P*. The phosphorylation is reversible, the reverse action (transition *Dephos_IR_I_P*) correlates with the dissociation of insulin. Alternatively, the activated receptor-ligand complex can be internalized (transition *Inter_IR_I_P*). The phosphorylated complex in cytosol (place *IR_I_P_Int*) can be either transported back to the membrane (transition *Deinter_IR_I_P*) or deactivated in the cell (transition *Dephos_IR_I_P*). The inactive unbound receptor in the cytosol can be transported back to the membrane (transition *Deinter_IR*) or be destroyed by degradation (transition *Degradation*). A process of synthesis sustains the level of IR.

#### Simulation of the model

The model is simulated using the Gillespie’s algorithm. Model parameters (initial concentrations of IR and reaction rate constants) are adapted from [35]. The volume of the system is set to 1E-9 l. Initial concentrations of the places are listed in Table 3 and the mass action reaction rate constants in Table 4. The rate of IR synthesis depends on the concentration of the receptor in the cytosol. If the concentration decreases below the steady-state value of 1E-13 M, an increased synthesis rate is used. This is described by the expression

**Table 3:** **Places of the Petri net of the IR model and the initial concentrations of the modeled compounds**

**Place**	**Initial concentration**
Insulin	varying
IR	9·10^−13^ *M*
IR_I	0
IR_I_P	0
IR_I_P_Int	0
IR_Int	1·10^−13^ *M*

**Table 4:** **Transitions of the Petri net of the IR model and the rate constants of the modeled reactions**

**Transition**	**Reaction rate constant**
Bind_Insulin	1·10^6^ *M* ^−1^ *s* ^−1^
Diss_Insulin	3.33·10^−3^ *s* ^−1^
Inter_IR	5.56·10^−6^ *s* ^−1^
Phos_IR_I	41.66*s* ^−1^
Dephos_IR_I_P	3.33·10^−3^ *s* ^−1^
Inter_IR_I_P	3.5·10^−5^ *s* ^−1^
Deinter_IR_I_P	3.5·10^−6^ *s* ^−1^
Dephos_IR_I_P_Int	7.68·10^−3^
Deinter_IR	5·10^−5^ *s* ^−1^
Degradation	2.783·10^−6^
Synthesis	2.78·10^−19^ *M*·*s* ^−1^
	if *I* *R*_*I* *n* *t*+*I* *R*_*I*_*P*_*I* *n* *t*≥10^−13^ *M*
Synthesis	1.67·10^−18^ *M*·*s* ^−1^
	if *I* *R*_*I* *n* *t*+*I* *R*_*I*_*P*_*I* *n* *t*<10^−13^ *M*



We simulated two days with a typical 24 h insulin profile of a healthy person, whereat the mean basal insulin concentration is about 6·10^−11^ M [36]. Meal intake, simulated at 09:00 h, 13:00 h and 18:00 h, triggers the rapid increase of the insulin concentration to approximately 3.6·10^−10^ M [36]. The hormone concentration returns to the basal level within the next 3 hours. This profile is described by a mathematical expression with four cases (one condition-free case for the basal concentration and three time-dependent cases for the meal intakes) and a negative exponential function which describes the decrease of insulin concentration after meal intake. The expression can be found in the supplementary text file “example_MathExp” (Additional file 3).

**Figure 4:**
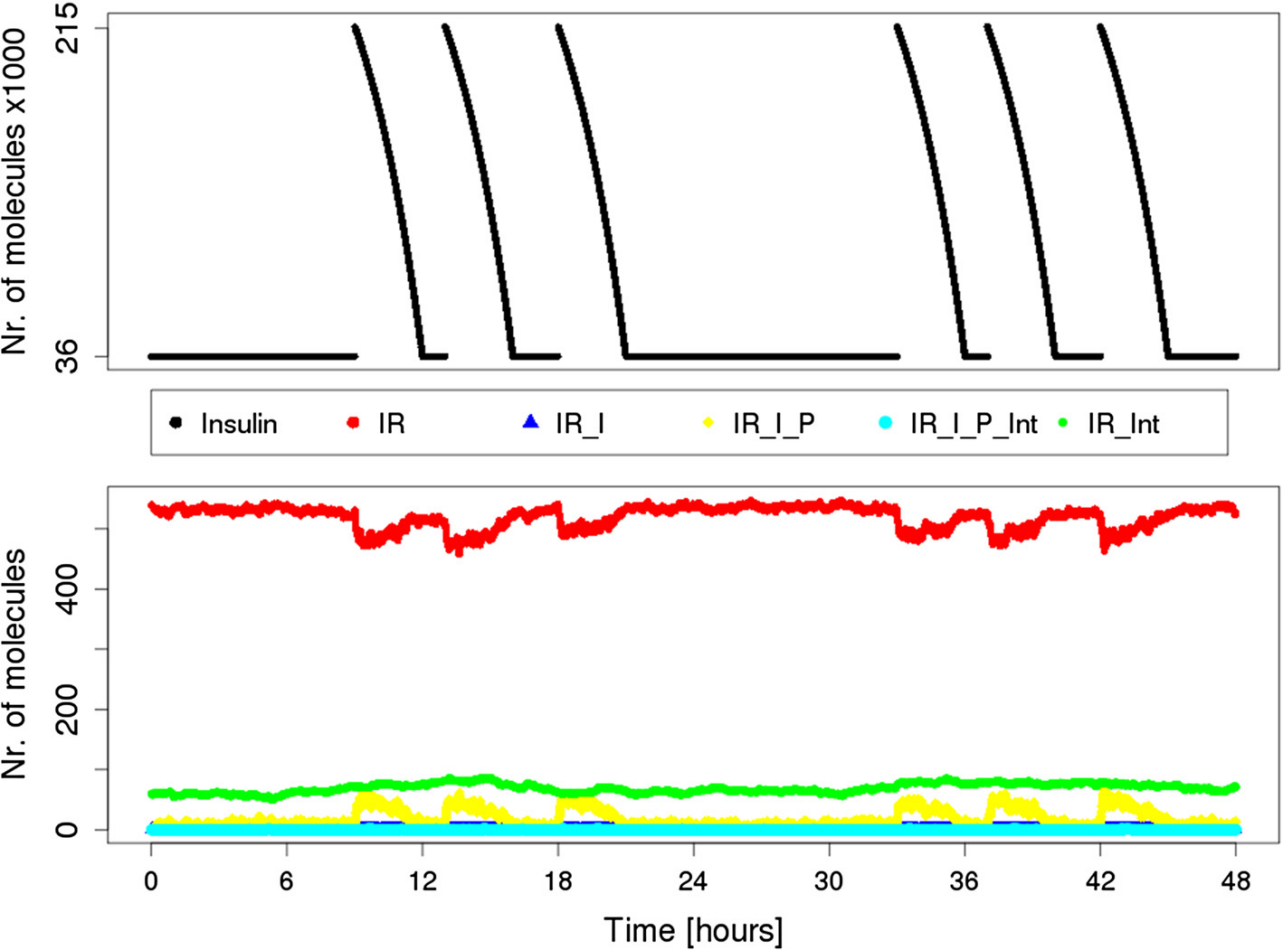
Results of a 48 h simulation of the IR model. The IR cycling model was simulated for two days with a typical insulin profile. The basal insulin level is 6·10^−11^ M. At 09:00 h, 13:00 h and 18:00 h, meal intake stimulates insulin secretion and leads to a peak postprandial concentration of 3.6·10^−10^ M which returns to its basal state within three hours. The numbers of insulin molecules (upper part) and the number of molecules of different states (lower part) are plotted against the simulated time. Black points represent insulin, red points the free membrane located receptor, blue points the receptor-insulin complex (inactive), green the free receptor in cytosol, yellow the activated phosphorylated IR-insulin complex in cytosol and cyan the phosphorylated receptor-insulin complex in the membrane.

Results of a simulation of two days are plotted in Figure 4. The figure shows on top the number of insulin molecules plotted against time. Whereas the number of molecules of different IR states is depicted at the bottom. The PN (Additional file 4) is provided as the MONALISA-project file IR_Model.mlproject. The simulation setup file “IR_Model_params.xml”, (Additional file 5) can be found in the supplement.

## Conclusions

MONALISA combines the powerful modeling concept of Petri net formalism with stochastic simulation abilities implementing a simulation module. The intuitive graphical user interface allows to focus on modeling and simulation. Different simulation modes make the software suitable for working with PN in various areas, especially for biological tasks like analysis and simulation of metabolic systems, gene regulatory networks or signal transduction pathways. Constant places and the usage of mathematical expressions for describing simulation parameters offer an improved flexibility compared to other tools, allowing for modeling of non-standard kinetics and complex relationships to the external environment. Useful features, like built-in plotting, navigation through the simulation history, export of the simulation setups to XML-files and setting the seed of the random number generator, can help to easily adjust parameters to follow and assess simulation results. We demonstrate a small case study that models the prominent motif of the recycling of the insulin receptor. The case study presents the abilities of MONALISA to model and simulate complex biochemical systems and demonstrates the applicability of the software for studying biological processes.

## Availability and requirements

**Project name:** Simulation mode for MONALISA - a tool for development, visualization and analysis of Petri nets**Project home page:** http://www.bioinformatik.uni-frankfurt.de/tools/monalisa/ **Operating system(s):** Linux, Windows, MacOS**Programming language:** Java, C (for invariantscomputation)**Other requirements:** Java 1.7 or higher**License:** Artistic License 2.0**Any restrictions to use by non-academics:** None

## Additional files

Additional file 1
**Current version of** MONALISA. The current version of MonaLisa, including the implemented simulation module, is provided as executable.jar-file as well as source files. To execute the compiled file, open a console (command line under Windows or terminal under UNIX-like systems), navigate to the directory where the MonaLisa.jar is located and type java -jar MonaLisa.jar.

Additional file 2
**Documentation of the Simulation Mode of** MONALISA. Documentation of the simulation mode.

Additional file 3
**Mathematical expression of an insulin profile.** The text file “example_MathExp” contains the mathematical expression which is used for modeling a 24h profile of insulin concentration in healthy patients with three meal intakes.

Additional file 4
**MONALISA** **-project of the insulin receptor recycling model.** The Petri net of the IR recycling model is provided as the MonaLisa-project file “IR_Model.mlproject”. To open it, start MonaLisa and select “Open project”.

Additional file 5
**Simulation setup.** Simulation setup, which includes initial concentrations of compounds and reaction rate constants, is stored in the XML-file “IR_Model_params.xml”. To load it, open the IR_Model - project file, start the “Gillespie SSA”-mode from the “Simulation”-tab and click the “Load setup”-button.

## Abbreviations

ODE: Ordinary differential equation
PN: Petri net
CME: Chemical master equation
SSA: Stochastic simulation algorithm
PRNG: Pseudo random number generator
IR: Insulin receptor
*r*_*i*_: Rate of reaction *i*
*k*_*i*_: Deterministic rate constant of reaction *i*
*c*_*i*_: Stochastic rate constant of reaction *i*
M: Molar concentration = moles per liter
*V*: Volume
l: Liter
*n*_*X*_: The number of molecules of compound X
[*X*]: Concentration of the compound X (or of the compound represented by the place X)
*N*_*A*_: Avogadro constant = 6·10^23^
