# Erratum to: MONALISA for stochastic simulations of Petri net models of biochemical systems

**DOI:** 10.1186/s12859-015-0725-7

**Published:** 2015-11-05

**Authors:** Pavel Balazki, Klaus Lindauer, Jens Einloft, Jörg Ackermann, Ina Koch

**Affiliations:** Department of Molecular Bioinformatics, Institute of Computer Science, Cluster of Excellence “Macromolecular Complexes”, Johann Wolfgang Goethe-University Frankfurt am Main, Robert-Mayer-Straße 11-15, Frankfurt am Main, 60325 Germany; Sanofi Aventis Deutschland GmbH, Industriepark Höchst H831, Frankfurt am Main, 65926 Germany

Erratum

After publication of the original article [1] the authors have brought to our attention that the following revisions had not been incorporated into the final published version.

In Definition 1 (Petri net): “E ⊆ ((P x T)∪(T x P))” is the set of directed edges not “E ⊆ ((P T) ∪ (T P))”. A segment of the Legend in Figure two: “A model of insulin receptor activation and recycling.” was incorrect and has been removed. The Legend of Figure three was incorrect and the correct legend is: “The model of insulin receptor recycling according to Figure 2 is represented as a Petri net. Places are drawn as circles and transitions as black squares.”

These mistakes have been updated in the original article as detailed in this erratum.
