# Supplementary material for: MONALISA for stochastic simulations of Petri net models of biochemical systems
Source: BMC Bioinformatics. 2015 Jul 10;16:215. doi: 10.1186/s12859-015-0596-y (PMC4496887; doi:10.1186/s12859-015-0596-y)
Supplement: Additional file 2 — Documentation of the Simulation Mode of MONALISA. Documentation of the simulation mode. [file 12859_2015_596_MOESM2_ESM.pdf]

Simulation mode of MonaLisa - Documentation

Version 1.1

Written by Pavel Balazki and Jens Einloft
December 19, 2014

If you find any errors in this document, please contact pavel.balazki@gmail.com.
If you find any errors in the software, please report them using the SourceForge ticketing system (`http://sourceforge.net/p/monalisa4pn/tickets/`) or sending an email to Einloft@bioinformatik.uni-frankfurt.de (general MonaLisa problems) or pavel.balazki@gmail.com (simulation specific problems).

Abstract

Apart from creation, visualization and static properties analysis of Petri nets, MonaLisa allows dynamic simulation of net behavior, ranging from simple random transition firing to complex and comprehensive stochastic simulation of biochemical networks. A user-friendly graphical interface, combined with interactive visualization of simulated events, provides access to various simulation parameters and allows the user to monitor and analyze the simulation outcome in real time as well as export of simulation results into a text file for further examination. Some of the key features of the software are visualization of the simulation by drawing the number of tokens on the places, interactive plotting, export of results into text files, mathematical expressions for describing simulation parameters and an extensive stochastic simulation mode for systems of chemical reactions (Stochastic Simulation Algorithm, SSA). The combination of the powerful concept of Petri nets, intuitive graphical user interface and high-performance simulation abilities makes MonaLisa a good choice for scientists in systems biology.

The first part of this document explains the functionality of the simulation mode of MonaLisa, guiding the user through particular elements of the software. The second part describes the four implemented simulation modes in detail. The third part contains the description of the implementation of some parts. The fourth part gives an example of modeling and simulation of a biochemical system of insulin receptor recycling, a system which is of high importance because of its involvement in diseases like the metabolic syndrome and Type II Diabetes mellitus. The final part lists and explains all elements of the user interface. It is assumed that the user is familiar with the Petri net concept and the basic functions of MonaLisa.

Contents

1 Simulation mode - step-by-step guide 4
1.1 Starting a simulation 4
1.2 NetViewer in simulation mode 5
1.3 Constant places 6
1.4 Performing simulation steps 6
1.5 History and snapshots 8
1.6 Custom markings and simulation setups 8
1.7 Plotting results 11
1.8 Statistics 11
1.9 Preferences 12
1.9.1 Writing simulation results into a text file 12
1.9.2 Snapshots and other preferences 13
1.10 Using mathematical expressions 14
1.10.1 General usage 14
1.10.2 Supported syntax 16
1.10.3 Conditional expression 16

2 Different simulation modes 17
2.1 Asynchronous 17
2.2 Synchronous 17
2.3 Stochastic 18
2.4 Gillespie SSA 21
2.5 Fast simulation mode of the Gillespie SSA 22
2.5.1 Using fast simulation mode 23

3 Implementation details 26
3.1 Structure of the XML file for simulation setups 26
3.2 Random number generator 27
3.3 Drawing waiting times in stochastic mode 28
3.4 Gillespie SSA implementation 28
3.4.1 Converting input data 28
3.4.2 Performing a simulation step 29
3.5 Exact SSA of the fast mode 31
3.6 Approximate SSA of fast mode 31

4 Simulation of insulin receptor (IR) recycling model - a practical example 32
4.1 Insulin receptor model 32
4.2 Simulating the model 33

4.3 Comparing Exact and Approximate SSA 35

5 Elements of the GUI **36**

5.1 “Simulation”-tab of the ToolBar 36

5.2 “Simulation preferences”-window 38

5.3 NetViewer in simulation mode 39

5.4 Synchronous mode - specific controls 40

5.5 Stochastic mode - specific controls 40

5.6 Gillespie SSA - specific controls 41

5.7 Fast simulation mode 41

6 License **43**

7 Frequently Asked Questions **43**

8 Changelog **44**

9 Acknowledgments **44**

1 Simulation mode - step-by-step guide

This section explains the features of the simulation mode, from starting the simulation through changing preferences to exporting results. Users who are unfamiliar with the program will learn how to work with it, and more experienced users may find interesting details about various functions. The general description of MonaLisa can be found at http://www.bioinformatik.uni-frankfurt.de/tools/monalisa/.

1.1 Starting a simulation

To start a simulation, load an existing MonaLisa-project or create a new one from the main window of the MonaLisa-software. Select the **"Show NetViewer"**-button to open the graphical representation of the net and the ToolBar. Switch to the **"Simulation"**-tab in the ToolBar (see figure 1).

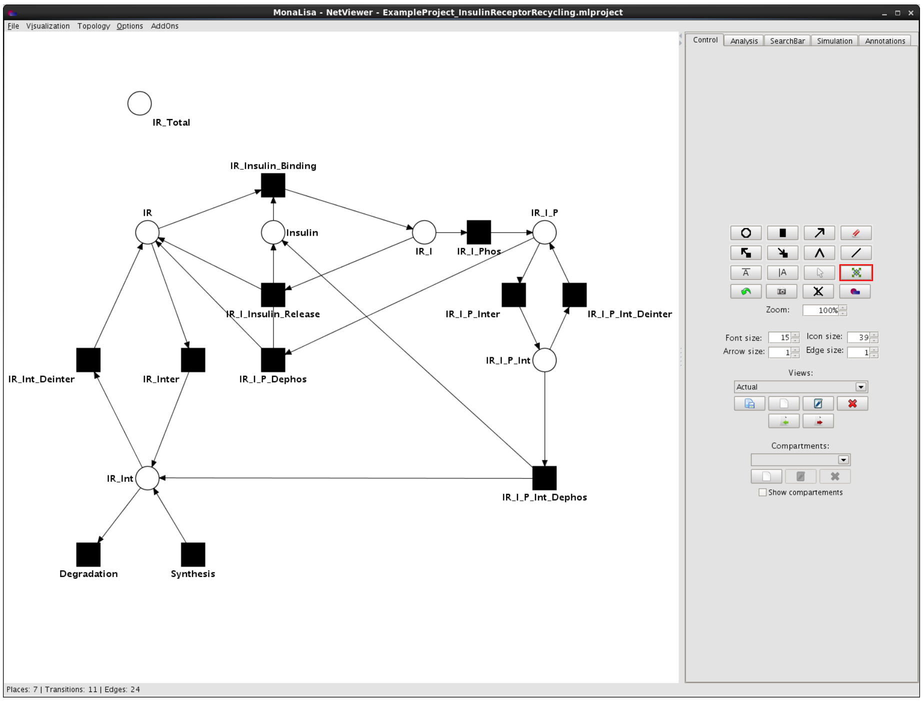

Figure 1: A Petri net opened in NetViewer with selected Simulation-tab from the ToolBar.

Now, select a simulation mode from the same-named drop-down menu and click on the **"Start simulation"**-button. There are four simulation modes implemented, each of which evolves the system in its own way. Simulation modes will be described later on (section 2). If you want to simulate a system of chemical reactions, select **"Gillespie SSA"** (described in section 2.4).

When the simulation mode becomes activated, it is impossible to edit the net structure. You can adjust font, icon, arrow and edge size with the corresponding field under the **"Start simulation"**-button. To return to the editing mode, press **"End simulation"**-button. It will deactivate all simulation controls and stop any running simulation. In the following sections common functionality of simulation mode will be explained using the **"Asynchronous"**-mode.

1.2 NetViewer in simulation mode

In simulation mode, mouse actions and visualization of net nodes are adjusted to provide additional controls and useful information about the simulation. The **number of tokens on a place** is represented either by red dots (token number 1 – 4) or by a digit (more than 4 tokens). Transitions have differently colored borders depending on their state. **Inactive transitions** are represented as black squares. **Active transitions**, i.e. those which can fire, have a **green border** or **green filling** (if the transition fired last and is still active). Transitions which fired last have a **red border**, hence transitions which fired last and are active have a **red border with green filling** (see figure 2).

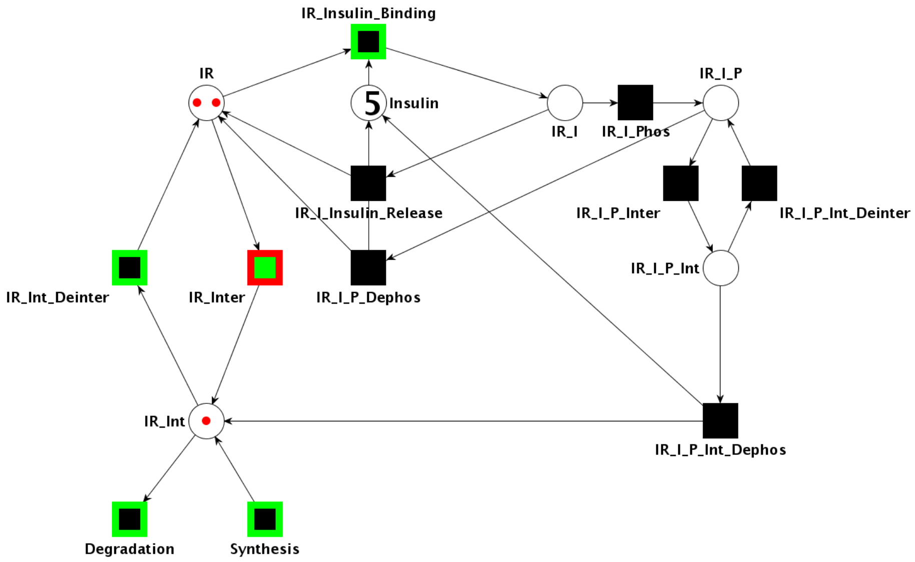

Figure 2: Representation of the Petri net in simulation mode. Active transitions have a green border or green filling. Last fired transitions have a red border. The number of tokens on a place is represented either by red dots or by a digit.

One can **fire an active transition** by clicking on it with the left mouse button. Selecting a **place** and opening the context menu with the right mouse button allows to input the number of tokens for the place or flagging the place as **constant** (constant places are described in the next section).

1.3 Constant places

One specific feature of the simulation mode in MonaLisa are the **constant places**. In contrast to "normal" places, the number of tokens on a constant place can not be changed by firing of transitions. According to the standard rules of Petri net, firing of a transition consumes tokens on the pre-places of the transition (the number of consumed tokens is given by the weight of the arc between the pre-place and the transition) and adds tokens to the post-places, according to the arc weight. The constant places, however, are ignored in the firing step, although they are still considered while determining which transitions are active. This feature can be useful when modeling input conditions which are not affected by the modeled system. For example, in the model of insulin receptor recycling (the model is briefly described in section 4) insulin is modeled as a constant place. It is assumed that insulin concentration is maintained and regulated by processes which are not modeled, and a single cell (which is modeled with the net) does not affect its global concentration.

Furthermore, the number of tokens on a constant place can be described by a complex mathematical expression. For instance, pulsatile insulin secretion can be described as

$$(1400 \times cos(Time/300 \times 2\pi) + 1600)$$

where the variable *Time* stands for the simulated time (in seconds). For more information how to use mathematical expressions, see section **"Using mathematical expressions"** 1.10.1.

If a non-constant place carries tokens and is selected to be constant, it becomes a mathematical expression with the value of the token numbers assigned. If a constant place with a mathematical expression is selected to be non-constant, its expression is evaluated and the number of tokens is assigned to the non-constant place.

1.4 Performing simulation steps

As mentioned above, you can fire an active transition by clicking on it with the left mouse button. The other way of performing simulations is by specifying the number of simulation steps and letting the algorithm perform them by the selected rules. The controls are located in the area below the **"Start-"** and the **"End simulation"**-buttons (figure 3). On top of this area is the name of the selected simulation mode. You can specify the number of simulation steps in the text area **"Steps to perform"**. Alternatively, you can enable the check box **"Continuous mode"** if the simulation should go on until no active transitions

left or is interrupted otherwise.

After the number of steps is selected, click on the **“Start simulation sequence”**-button. This will start firing sequence of the specified number of steps. The button turns into the **“Stop simulation sequence”**-button, clicking on which terminates the ongoing simulation sequence.

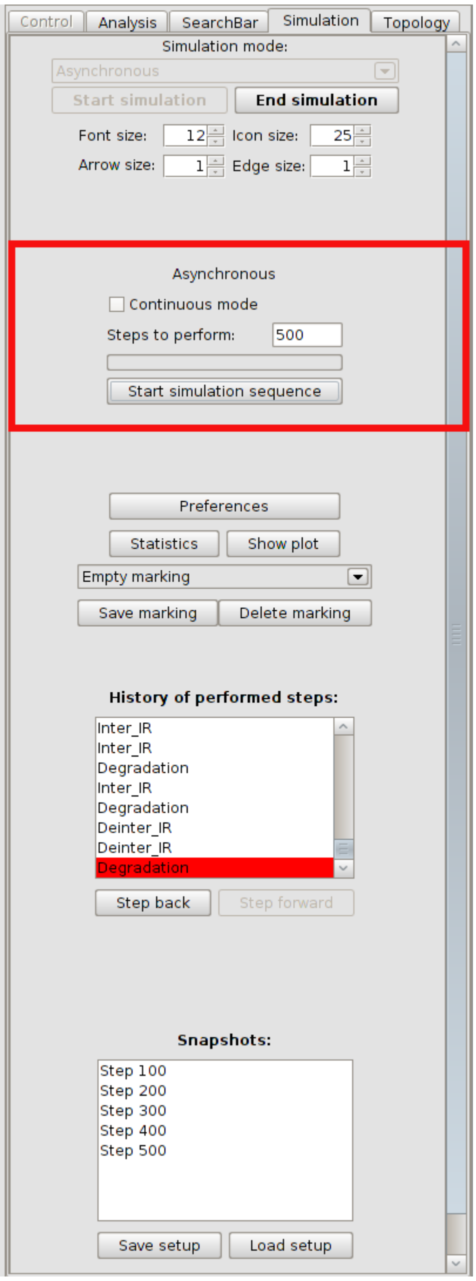

Figure 3: Defining the number of steps to simulate.

1.5 History and snapshots

The software tracks the simulation in a history list. The area **"History of performed steps"** (figure 4) shows which transitions have fired in the last 100 performed steps. The step performed last is highlighted with red background. If the list is full (shows 100 steps) and a new step is performed, it is written to the end of the list, and the first element is removed. You can navigate through the history by selecting a step with the left mouse button or clicking the buttons **"Step back"** and **"Step forward"**. The visual output in the NetViewer will be updated to the system's state at the selected step. Firing transitions which are not part of the history (e.g. by selecting a transition from the NetViewer or starting a new simulation sequence) will erase all history steps following the last selected step.

By default, a **snapshot** of the system is taken after each 100 steps. A snapshot contains the system's state at the corresponding step and the history of the last 100 steps. By selecting a snapshot from the **"Snapshots:"**-list, its history is loaded into the history area. The consequence is that though the history can only track the last 100 steps, selecting snapshots allows the user to have a complete history of the simulation, giving the possibility to return to every single state occurred during the simulation. Though the shown history can be erased by firing transitions (e.g. if you select the second step from the history and fires a new transition, steps 3-100 will be erased), it does not affect the snapshots.

Snapshots provide a detailed insight into the simulation sequence on the cost of increased memory consumption. For large simulations (several hundreds or thousands of steps) and low memory configurations, it may be advisable to turn snapshots off in the **"Preferences"**-panel (section 1.9). You can also turn snapshots on and off between the simulation sequences.

1.6 Custom markings and simulation setups

A state of the simulated system is represented by its marking. The program provides a useful option of saving and loading markings (figure 5). You can save the current state at any point by clicking on the **"Save marking"**-button. This marking can then be restored by selecting it from the drop-down menu. The currently selected marking can be removed with the **"Delete marking"**-button. By default, each net is provided with the "empty marking" (no tokens are allocated on the places). Upon saving a marking, mathematical expressions of constant places are evaluated, and the values are stored as numbers. When restoring a marking by selecting it from the menu, only values of non-constant places are adopted, those of constant places are ignored. If you want to load

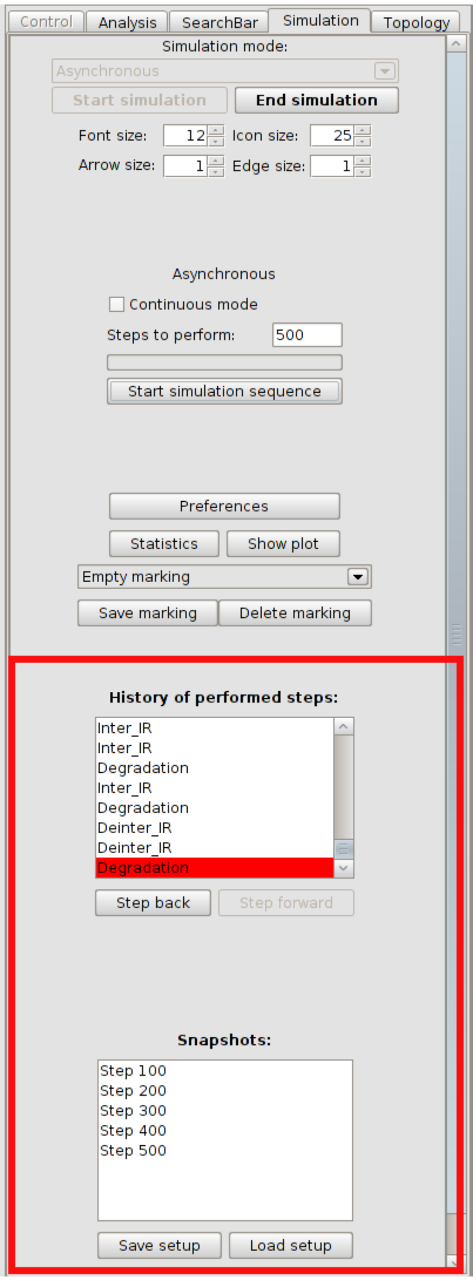

Figure 4: History and snapshots.

a value of a constant place from the marking, you must first set the place to non-constant by right-clicking on it and de-selecting the **"Constant token number"**-check box. The marking only stores the actual number of tokens on places and cannot be used for storing mathematical expressions. Applying a custom marking also clears the history and the snapshots list. All custom mark-

ings are saved with the **view** (see NetViewer documentation) and are available next time the project is loaded.

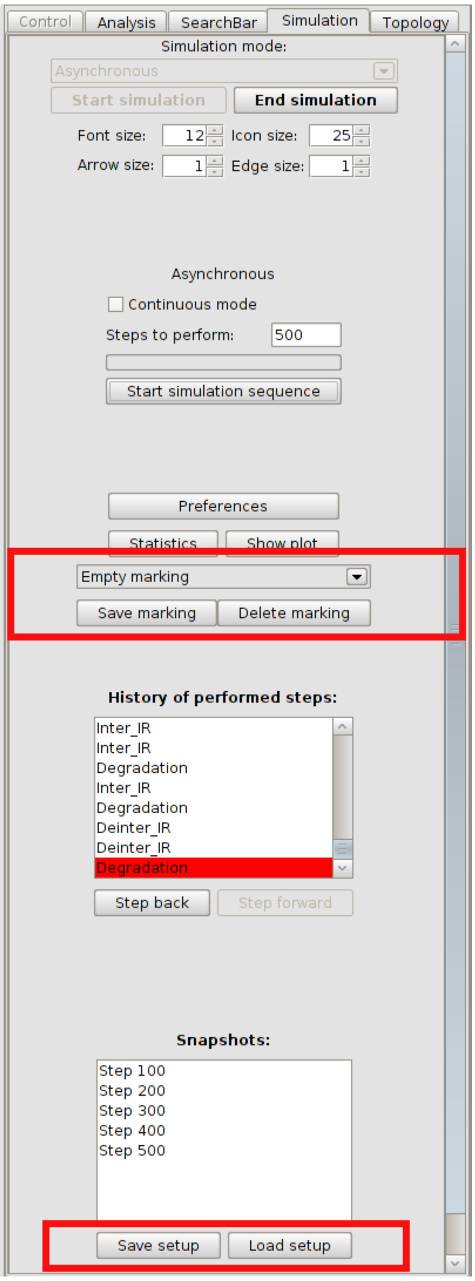

Figure 5: Custom markings and simulation setups.

The option of saving more information about the simulation setup, such as mathematical expressions for constant places or firing rates of transitions, is provided by creating an XML file with setup parameters by clicking on the **“Save**

setup"-button. To load a valid simulation setup, click on the **"Load setup"**-button and choose a file which stores the settings. The structure of the setup files is described in section 3.1. The program tries to load all possible settings from the selected file. The settings of the places and transitions are mapped to the IDs, so it is possible to rename the nodes or create new ones without losing the compatibility with a setup file. The information stored in the setup file depends on the simulation mode and the supported features. Unsupported features are simply ignored upon loading.

1.7 Plotting results

The simulation mode of MonaLisa provides plotting functionality with features like zooming or exporting plots to PNG-format files. To plot the results of your simulation, click on the **"Show plot"**-button (see figure 6). A window called **"Results plot"** shows up. It plots the number of tokens on the places against the simulated time. It is possible to zoom in or out and to export the plot into a PNG file by right clicking on the image and opening the context menu. You can also zoom in a region by selecting it with the mouse or using the mouse wheel.

Storing results for plotting is memory-consuming, so it might be advisable to disable this feature for long simulations if it is not needed. This can be done in the **"Preferences"**-window by un-checking the **"Built-in plotting"**-check box. You can also select the places which results should be plotted with the **"Places to plot"**-button.

1.8 Statistics

Click on the **"Statistics"**-button (highlighted in figure 7) to open a window with some statistic information about the current simulation. There, you can see the number of simulated steps and how often each transition has fired. In the lower half of the window you can see a list of the steps for which statistics is available. A statistic is recorded for the step at which the window was created and, if snapshots are enabled, for each snapshot. Selecting a step from the list displays the statistic data of it. Finally, at the bottom of the window you can see the number of simulated steps and the number of fired transitions at the selected step. The number of fired transitions can be higher than the number of simulated steps since some simulation modes (e.g. synchronous) allow multiple transition firings per step.

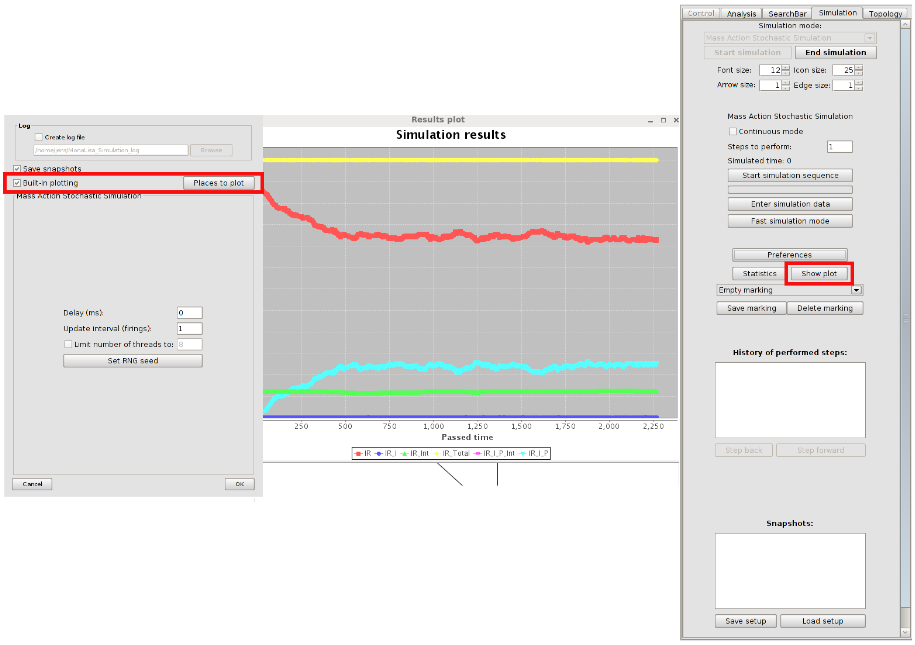

Figure 6: Plotting simulation results.

1.9 Preferences

To adjust advanced simulation parameters, click on the **“Preferences”**-button. This will open a new window with control elements (see figure 8).

1.9.1 Writing simulation results into a text file

To write simulation results into a file, select the **“Create log file”**-check box. You can now select the destination path of the file by entering it in the text field below or by clicking the **“Browse”**-button. A text file is created upon first transition firing. For each simulation instance (initiated by starting of a simulation mode), a separate file is created with the name **“Sim_log_-[currentDateAndTime].csv”**.

The file consists of elements separated by tab stops and line breaks. The first row contains the names of the places. The second row contains the IDs of the nodes of the corresponding places. The third row is a separation placeholder “————”. From the fifth row on, the first element is the number of simulated steps, the second element is the simulated time, the third are the names of the transitions fired in this step (separated by a semicolon), and the following elements are the number of tokens on the places. Be aware that elements are separated by tabs only and not by semicolons, since some spreadsheet programs will try to use semicolons as separators, too.

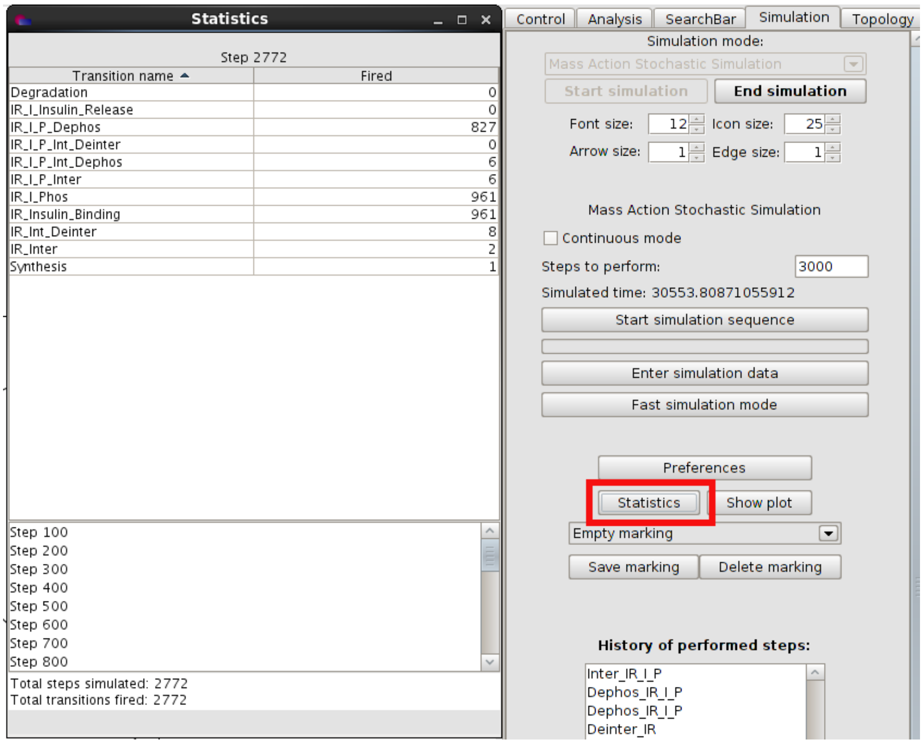

Figure 7: Statistics window.

1.9.2 Snapshots and other preferences

You can activate or deactivate snapshots (see section 1.5) by checking or unchecking the **"Save snapshots"**-check box.

The area below contains further parameters provided by the particular simulation mode. Common parameters are the delay between firings and the update interval. In the **"Delay (ms)"**-text field you can specify how long the simulator will wait before performing the next step. Zero stands for no delay, i.e. the next firing will occur as soon as the previous was processed. You can use delays for better visual tracking of the simulation progress.

The area **"Update interval (firings):"** controls the frequency of visual update in the NetViewer. For simulations with a huge number of simulation steps it can be useful to update the visual output not after each step (default value 1). 0 stands for no update at all. However, after the simulation sequence is finished, the visual output is always updated. Updating visual output costs execution time. Disabling updates or invoking them less frequent can speed up a simulation with a large number of steps.

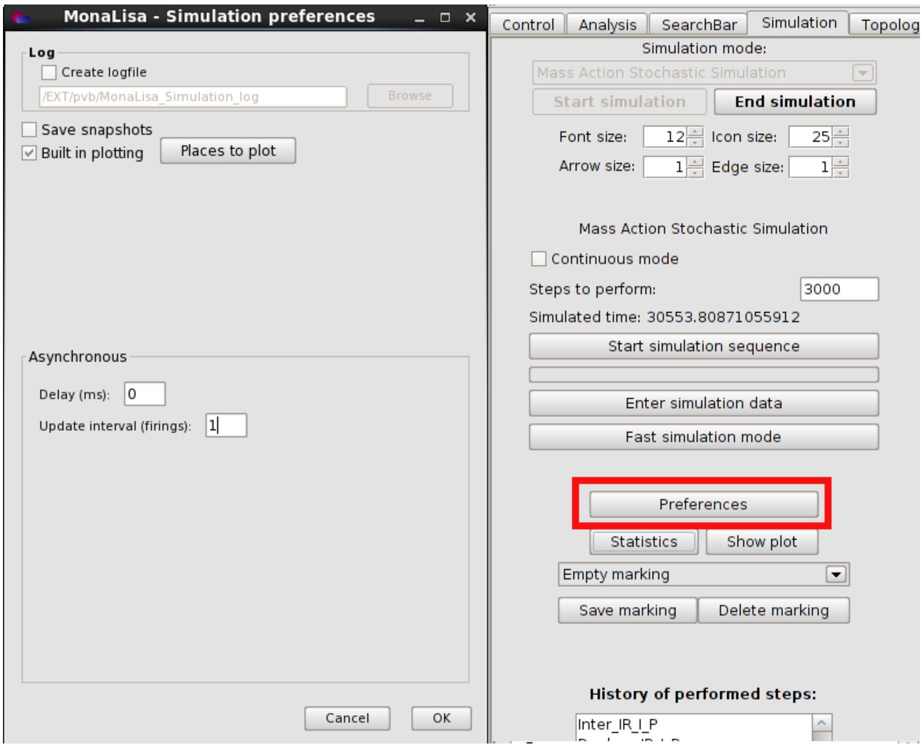

Figure 8: Preferences window.

1.10 Using mathematical expressions

1.10.1 General usage

Mathematical expressions provide a simple but flexible functionality of describing the number of tokens on constant places or reaction rate constants in the **"Gillespie SSA"**-mode. You can write expressions and functions which will be evaluated at each simulation step. These expressions can contain standard operators and some build-in functions and use *non-constant place's names* or the *simulated time* as variables.

As example, figure 9 shows the process of entering a mathematical expression for a constant place. To bring up the shown dialog, open the context menu of a constant place (by selecting a place and right clicking on it) and choose **"Set the number of tokens for this species"**. The expression is entered into the upper left text area. In this example, the place **"IR_Total"** carries the total number of insulin receptor (IR) molecules. For this, the number of tokens on this place is expressed as the sum of tokens on the places which represent different states of the insulin receptor:

`IR + IR_I + IR_Int + IR_I_P_Int + IR_I_P`.

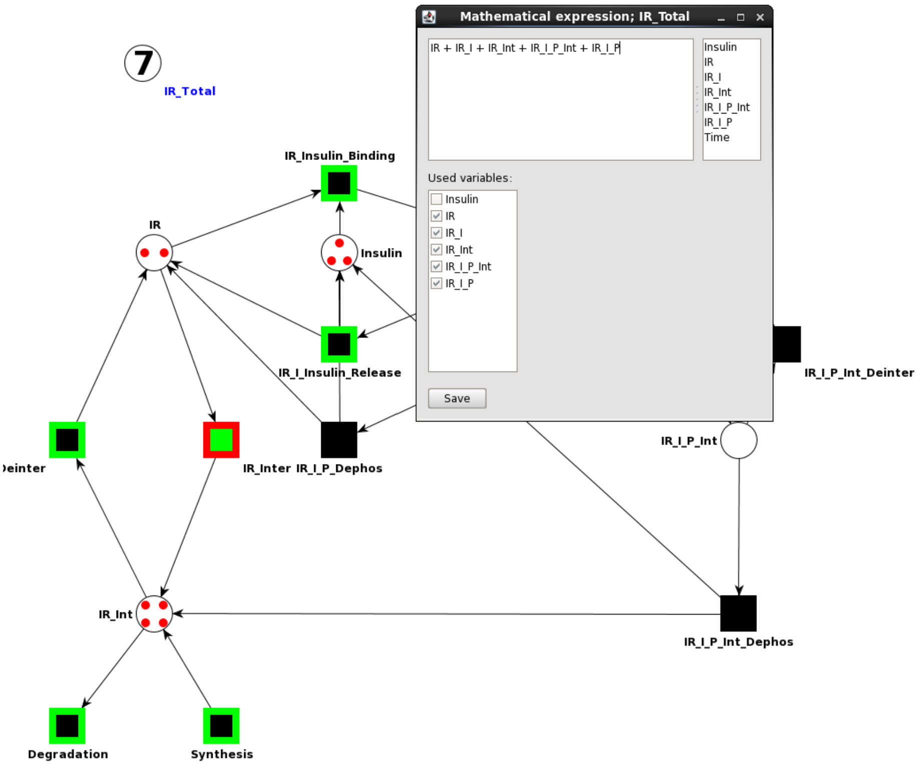

Figure 9: Entering mathematical expression.

Names of non-constant places are listed in the area to the right and can be inserted into the expression by clicking on them. You can also use the simulated time by selecting the variable **"Time"**. For the simulation modes "synchronous" and "asynchronous", the time equals to the number of simulated steps. For timed PN simulation in the "stochastic" mode, the time is measured in arbitrary units, for the "Gillespie SSA" the units are seconds.

An expression must *know* which variables it uses to be able to map the names of the places to the number of tokens they carry. Places used as variables are listed in the **"Used variables"**-list located below the expression-area and are checked. Every time the user selects a place from the right list and inserts it into the expression, a corresponding check box becomes activated. If you enter the name of the place without selecting it from the list, you *have* to enable it in the **"Used variables"**-list, otherwise the expression will not be evaluated. It is, however, *strictly advised* only to select places which are actually used in the expression and uncheck those which are removed from it. The reason is that the more variables an expression uses, the more expensive it can become to compute a simulation step. Furthermore, it is not allowed to use constant

places as variables to prevent an unsolvable reference cycle.

1.10.2 Supported syntax

The evaluation of a mathematical expression is based on the free Java library exp4j [2]. It supports the numerical input in standard and scientific notations (`1*10^2 = 1E2`) and various operators and functions, which are listed in table 1. Additionally, the variable **"pi"** can be used for the number $\pi = 3.14159\ldots$.

Table 1: Operators and functions which are supported by mathematical expressions for describing the number of tokens (or concentrations) on constant places or reaction rate constants.

**Operators**	
Addition	2 + 2
Subtraction	2 - 2
Multiplication	2 * 2
Division	2 / 2
Exponentiation	2 ^ 2
Unary Minus, Plus (Sign Operators)	+2 - (-2)
Modulo	2 % 2
**Functions (use as "func(x)")**	
abs	absolute value
acos	arc cosine
asin	arc sine
atan	arc tangent
cbrt	cubic root
ceil	nearest upper integer
cos	cosine
cosh	hyperbolic cosine
exp	Euler's number raised to the power (e^x)
floor	nearest lower integer
log	natural logarithm (base e)
sin	sine
sinh	hyperbolic sine
sqrt	square root
tan	tangent
tanh	hyperbolic tangent
div(x,y)	integer division, e.g. **div(28,24)** returns 1

1.10.3 Conditional expression

It is possible to use multiple conditional expressions to define the reaction rate constant or the number of tokens on a constant place. The condition syntax has the form

```
if [condition 1] and ... and [condition]
```

```
then [expression]
```

where `[condition]` is composed of the three parts:

```
[expression 1] [operator] [epxression 2]
```

with `[expression 1]` and `[expression 2]` are mathematical expressions with the above described syntax, and `[operator]` is one of the operators “=”, “<”, “>”, “<=” or “>=”. If all conditions are satisfied, `[expression]` is evaluated and returned as a value. Several conditional expressions can be separated by “;”. The value of the first conditional expression whose conditions hold is returned. If a case has no conditions, it is automatically valid (e.g. “5+3” is a simple case without conditions). If none of the described cases is valid, 0 is returned. For example, the number of insulin molecules should be 1000 for the first 5 minutes, 100 for the next 5 minutes and 0 afterwards:

```
if Time <= (5*60) then 1000;
if Time <= (10*60) then 100.
```

As long as the seconds’ count stays below 300, the first case holds and 1000 is returned. If the number of simulated seconds is greater than 5*60, but less than 10*60, the second case is evaluated. After that, no case holds and 0 is returned.

2 Different simulation modes

This section describes the four simulation modes which are implemented in MonaLisa in detail.

2.1 Asynchronous

Asynchronous is the common mode of a PN simulation. In this mode, one transition is fired per simulation step, and the firing does not consume any time. All active transitions have the same probability to be chosen to fire.

The setup file stores the information about the number of tokens on non-constant places and the mathematical expressions of the constant onces. The “Time”-column in the log file represents the number of simulated steps.

2.2 Synchronous

In the synchronous mode, multiple active transitions can fire in one step simultaneously. By default, the simulator tries to fire all active transitions at once.

Transitions which share pre-places are called *concurrent* and compete for the tokens of the shared pre-places. If the number of tokens on a place is not sufficient for all post-transitions, transition(s) which should fire are chosen randomly with equal probability until all tokens are consumed.

In the **"Preferences"**-window the fraction of active transitions to fire per step can be selected. The standard value is 100%, lowering the value will result in firing only a fraction of active transitions. The actual number of fired transitions is rounded up, so selecting 50% of three active transitions will result in firing of two transitions. The transitions are selected randomly with equal probability in each step.

The setup file stores the information about the number of tokens on non-constant places and the mathematical expressions of the constant places. The "Time"-column in the log file represents the number of simulated steps.

2.3 Stochastic

Stochastic PN are a specific form of *transition-timed* PN. In a transition-timed net, a transition must wait a defined time before it can fire after it was activated. Generally, the units of time are not specified but can be modeled as seconds or other common time units. This technique allows to model systems with time-consuming processes, therefore timed PN are widely used for modeling biochemical reactions.

In stochastic PN, waiting times dt are chosen stochastically from the probability density function

$$f(dt) = r(T_i) \times e^{-r(T_i) \times dt}$$

with the firing rate $r(T_i)$ of transition T_i. The waiting times are exponentially distributed. A firing rate expresses how often (in average) a transition will fire in a single time unit. A transition with the firing rate $r(T_i) = 1$ will fire once every time unit, a transition with the firing rate $r(T_i) = 0.5$ once every second time unit and a transition with $r(T_i) = 2$ will fire twice in one time unit.

To specify the firing rates, click on the **"Firing rates"**-button in the "Stochastic" simulation mode. A window will show transitions and corresponding firing rates ordered in a table (figure 10). By default, all transitions have the firing rate 1. You can change the firing rate of a transition by double-clicking on it and confirming the new value with the "Enter"-key.

At each simulation step, the simulator determines the waiting time of each transition and fires the one with lowest waiting time. The **simulated time** (arbitrary units) is shown under the **"Firing rates"**-button and is actualized after every firing sequence (defined number of steps).

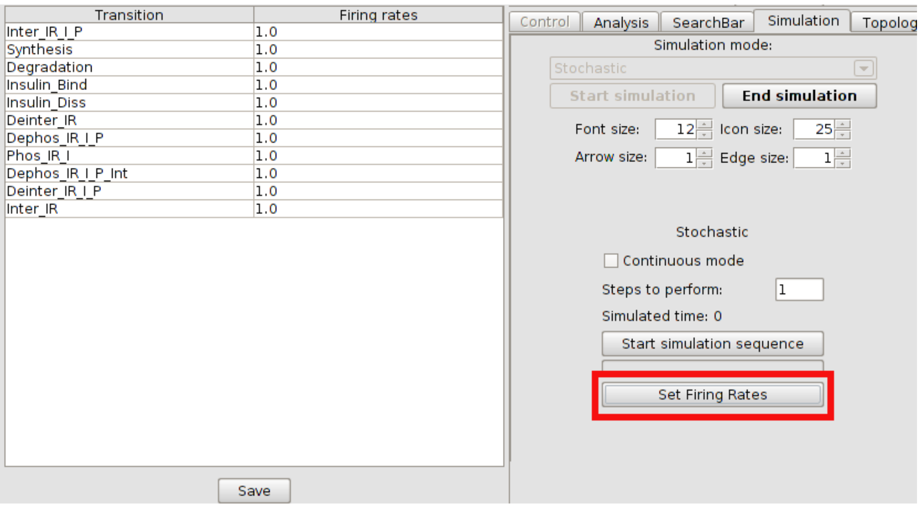

Figure 10: A window for specifying the firing rates of the transitions.

The firing rates of the transitions can be dependent on the current marking. This can be activated in the **"Preferences"**-window by selecting the **"Marking dependent firing rates"**-check box. In this case, the firing rate of transition T_i is multiplied by the number of possible firings $q(T_i)$. $q(T_i)$ describes how many firings the transition can perform at current marking before the tokens on the pre-places are depleted. The probability density function of the waiting times evolves to

$$f(dt) = q(T_i) \times r(T_i) \times e^{-q(T_i) \times r(T_i) \times dt}.$$

To mimic the stochastic behavior, waiting times are drawn using a *random number generator (RNG)*. Each simulation should be unique and the waiting times, and by that the sequence of the fired transitions, slightly different. This *randomness* is given by the "pseudo random" number sequences generated by the RNG. However, the sequence produced by the RNG is not really random and depends on the *"seed"*, a 48-bit long integer, of the generator. **Two simulations of the same parameters started from the same seed will always produce identical results.** The seed can be changed by clicking the **"Set RNG seed"**-button in the **"Preferences"**-window (figure 11). The current seed is shown in the appearing window. It is important to understand that simulation results can only be identical if their seeds were equal before any simulation step was performed.

The setup file stores the information about the number of tokens on non-constant places, the mathematical expressions of the constant places and the firing rates of the transitions. The "Time"-column in the log file represents the simulated time in arbitrary units.

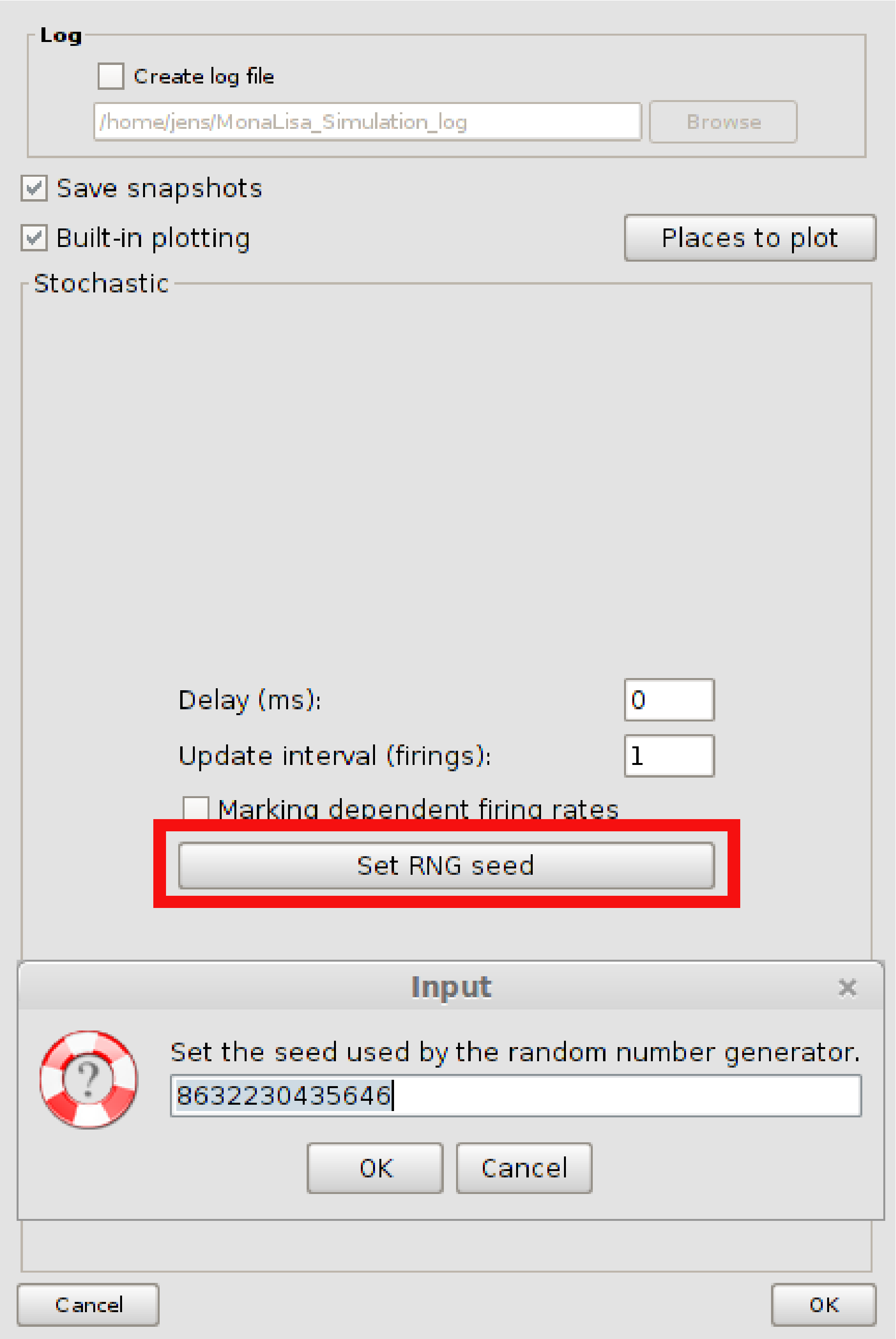

Figure 11: Setting the seed of the random number generator.

2.4 Gillespie SSA

This mode simulates the development of the numbers of molecules in a chemical system based on the mass action kinetic assumption. It is best fitted for single cell simulations. Stochastic simulation of chemical systems is an alternative to the widely-used Ordinary Differential Equations (ODE)-based modeling and was first described by Daniel Gillespie [3]. It allows to simulate occurrences of the chemical reactions of zero, first and second order.

The places of the PN model the chemical compounds (also in different states), the numbers of tokens represent the numbers of the corresponding molecules or complexes. Transitions represent the reactions, which have *reaction rate constants*. The actual reaction rate is given by the reaction rate constant and the number of the educt molecules. In each simulation step, the simulator decides *when* the next reaction will occur and *what* reaction it will be. For a detailed description of the simulation process, please see the original paper. Implementation details are given in section 3.4.

To perform a simulation, you must first provide the parameters of the system and the initial concentrations. For this, click on the **"Enter simulation data"**-button from the control area (figure 12). The data must be defined in the new opened window. In the first text area on the top of the window you must define the volume of the simulated system. The default value is 1 nanoliter which approximates the volume of an eukaryotic cell (of course, cells from different organisms and tissues can be much smaller or bigger). A large volume will result in the large molecule numbers and a slow simulation speed, so it is not advisable to perform whole-body simulations with several liters of medium.

The next table lists the places (i.e. compounds or compounds' states) of the system and their concentrations, given in mole per liter. Double-clicking on a concentration value allows to enter new values for non-constant places or opens a window for defining concentrations of constant places as mathematical expressions. The number of molecules, i.e. the number of tokens on the places, is computed automatically upon clicking on the **"Save"**-button.

Finally, rate constants must be defined for all reactions in the second table. The units are mole per liter and seconds; the concrete unit depends on the order of the reaction. For zero order reactions, the unit is $M \times s^{-1}$, for the first order s^{-1} and for the second order $M^{-1} \times s^{-1}$. New settings are applied by clicking on the **"Save"**-button. Closing the window without saving will discard all changes.

In the **"Preferences"**-window you can set the seed for the random number generator. See 2.3 for more details on the random number generator and seeds. Furthermore, the user can limit the number of parallel simulation runs. This

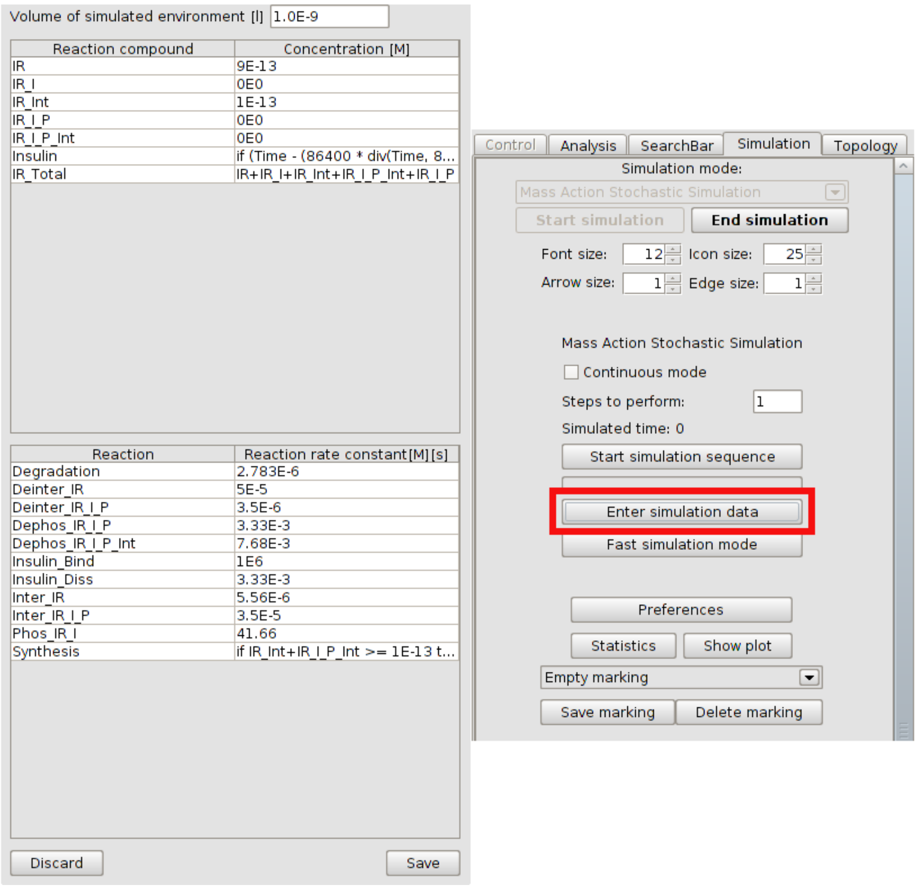

Figure 12: Input of simulation data.

option is valid for all running instances of the "fast simulation mode" (see next section 2.5). If the user limits the number of parallel runs and starts multiple instances of the "fast simulation mode", all runs are queued and processed as soon as new threads become available.

The setup file stores the information about the volume of the simulated system, the number of tokens on non-constant places, the mathematical expressions of the constant places and the mathematical expressions of the reaction rate constants. The "Time"-column in the log file represents the simulated time in seconds.

2.5 Fast simulation mode of the Gillespie SSA

The above described simulation mode makes use of all features of the simulator, including history, snapshots, plotting and step-by-step visualization. However, a simulation of biologically relevant chemical systems includes a large amount of

simulation steps, which makes the ability of tracking and visualization of each step not meaningful and the performance (expressed as the number of simulated reactions per time unit) becomes more important. The **fast simulation mode**, which can be started with the same-named button in the control area, offers a better performance by abstracting from the PN structure. It takes the input data from the PN based simulation mode and performs high-speed calculations with results' export into a CSV file. It allows up to 500 parallel simulations of the same scenario (with different outcomes due to the stochastic nature) and offers two algorithms - the *exact* stochastic simulation (equal to the one used in the PN simulation mode) and the *approximate* algorithm. In most cases, the approximate algorithm delivers similar results as the exact one, but much faster. The exact algorithm should be used for simulating systems with low molecule count, where stochastic effects have more significance.

2.5.1 Using fast simulation mode

To use the fast simulation mode, start the **"Gillespie SSA"** from the **"Simulation"**-tab of the ToolBar first. Enter the parameters of the system, like the volume, the initial concentrations of the compounds and the reaction rate constants, by clicking on the **"Enter simulation data"**-button or by loading an existing setup from an XML file with the **"Load setup"**-button. You can also set the seed of the Random Number Generator with the **"Set RNG seed"**-button from the **"Preferences"**-window. Any other setting, including the number of steps, snapshots or places to plot, will have **no effect** on the fast simulation mode. Clicking on the **"Fast simulation mode"**-button will bring the window of the fast simulation mode to the foreground (figure 13).

One important point that needs to be understood is that each new created fast simulation mode starts with the same seed of the RNG. That means that if you start two fast simulation modes from the same Gillespie SSA with the same settings, the outcome of the simulations will be identical. If you want a diversity in the results, you have to use different seeds. However, you must not change the seed each time by going to the preferences. The button **"New random"** in the fast mode window creates a new RNG with a new random seed.

In the upper text field you can specify the name and the location of the file for the results. The file consists of the elements which are separated by tab stops and line breaks. The first element of the first row is called "Step", the second "Time[sec]", the third "Reaction" and the following elements are the names of the places. The first element of the following rows counts the number of the simulated steps and the second element counts the simulated time. The third element is the name of the simulated reaction and the following elements are the

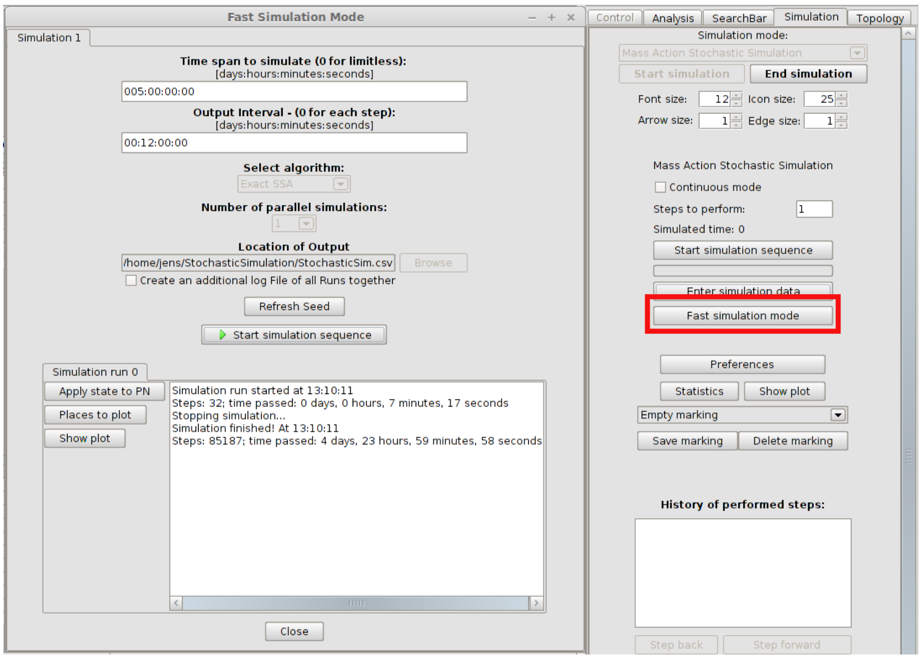

Figure 13: Window of the fast simulation mode with five parallel runs.

numbers of molecules of the corresponding compounds. This output file gives all the necessary information about the simulation and can be easily parsed by data analysis programs such as **R** or **MathLab**. While using the *approximate SSA algorithm* (see below), several reactions can occur per simulation step, and a single reaction can take place several times. In such a case, the third element of each row lists the names of all executed reactions, separated by a ";". However, this **does not** give any information about how often the reaction takes place.

In the drop-box **"Select algorithm:"** you can select the *Exact SSA* or the *Approximate SSA*. The exact algorithm simulates an occurrence of one reaction per step (described in [3]). This is the precise version of the SSA and provides a very close insight on simulation course, but is very time intensive for the larger systems or the high molecule counts. The approximate algorithm tries to speed up the simulation by *guessing* the number of reaction occurrences in a defined time step and executing several reactions per one simulation step (described in [4]). In most cases, the approximate algorithm provides a measurable speed increase compared to the exact one without significant precision loss on higher molecule counts. As a rule of thumb, one could say that for "low" molecule counts (up to 1000 for the most species) the exact method should be used. For the higher numbers of reactants the approximate method should be applied. Some comparisons of the methods can be found in the section 4.3.

The next drop-box allows to set up the **Number of parallel simulations** in the range from 1 to 500. Parallel simulation runs start with the same input data but different *seeds* and so allow the examination of the stochastic effects. The results are written in separate files, for each run an index is appended to the name specified in the **"Output"**-field. *Note:* The number of runs which are effectively executed parallel is limited to the number of cores available to the system (usually it would correspond to the number of cores of the machine). Remaining runs are queued and executed as soon as some of other runs are finished. Runs are considered as "finished" if the time given in **"Time span to simulate"** is simulated. You can also limit the number of parallel simulation runs (disregarding from which "fast simulation mode"-instance they are executed) in the **"Preferences"**-window.

In the text field **"Time span to simulate"** you can specify which time should be simulated. The value consists of four parts separated by a ":", which are (from left to right) days, hours, minutes and seconds. The simulation will run until the specified time is reached and will stop after that. If the field is left with the value "000:00:00:00", the simulation will run as long as there are reactions which can occur, or the user stops the simulation manually (identical to the **"Continuous mode"**).

By default, each simulation step is written into the output file. For long term simulations the number of steps can be very large, and the size of the output file becomes unmanageable. In the text area **"Write results into file interval:"** you can specify the interval of simulation time between writing the results. For example, if you select "00:00:00:01", the results will be written upon every simulated second. You can even change this value during the simulation (you need to stop the simulation, change the interval value and start the simulation again). If you wish, you can first simulate the system for 5 minutes (type "000:00:05:00" in the **"Time span to simulate"**-text field) with every step protocoled (leave the **"Write results into file interval:"**-text field with "00:00:00:00"). Let us assume that critical and rapid reactions happen during this time, and after that, the system is developing towards its steady state very slowly. You can now select the output interval to one second or even one minute and let the simulation run for further hours or days. In this case, you have both, a high resolution of the critical processes in the first 5 minutes and a manageable output file size because of the coarse resolution of the slower steady state development afterwards.

After having set up the simulation time, the update interval and the number of parallel runs, click on the **"Start simulation sequence"**-button. You can now see up to 500 tabs (depending on the selected number of the simulation runs) with the information about the simulation progress (figure 13), and the

button's capture changes to **"Stop simulation sequence"**. Clicking on it will terminate all simulation runs. The information in the big text field is updated every ten seconds and shows the number of the simulated steps and the simulated time. After the defined time has been simulated, the simulation stops and the button turns back to **"Start simulation sequence"**.

Each tab has three control buttons. The button **"Apply state to PN"** writes the numbers of the compound molecules to the marking of the PN. This is the convenient way to apply simulation results to the PN. The button **"Places to plot"** opens a window where you can select which places' values should be displayed in the plot. The plot is created with the **"Show plot"**-button and uses the values from the output file. That means that only results which were written into the file will be displayed.

It is possible to change the parameters of the system while a fast mode simulation is running and to start a new instance of the fast mode. You can even run further simulations of Gillespie SSA. It is, however, not allowed to end the Gillespie SSA and start another mode.

3 Implementation details

This section provides information about the implementation of some features and may be useful to advanced users.

3.1 Structure of the XML file for simulation setups

Simulation setups can be stored in an XML file, which has the following structure:

- **<SimulationSetup>**: root element, required.
  - **<volume>**: Element which stores the volume of the simulated system for the Gillespie SSA mode in a child text node.
  - **<places>**: Sub-elements store information about the places of the PN.
    - **<place>**: Element of a place. Has attributes *id* (integer value of the ID of the PN net node), *isConstant* (true or false, defines whether the place is constant), *name* (a string with the name of the place), *nrOfTokens* (an integer which describes the number of tokens on the place; only for non-constant places).
      - **<mathematicalExpression>**: If the place is constant, stores the mathematical expression which describes the number of tokens on the place.

        - <**expressionText**>: Text of the mathematical expression which describes the number of molecules on the place, stored in a child text node.
        - <**variable**>: Element of a variable of the mathematical expression. Each variable of a mathematical expression must be described in a <variable>-element! Has attributes *name* (a string with the name of the variable) and *placeId* (the ID of the place which represents the variable). Variables *pi* and *Time* need not to be specified.

- <**transitions**>: Sub-elements store information about the transitions of the PN.
    - <**transition**>: Element of a transition. Has attributes *id* (an integer of the ID of the PN node), *name* (a string with the name of the transition), *firingRate* (a double value which describes the firing rate of the transition. Used by the stochastic mode and ignored by other).
        - <**detReactionRateConstant**>: Rate constant of the reaction which is represented by this place. Used by the Gillespie SSA.
            - <**expressionText**>: Text of the mathematical expression which describes the reaction rate constant, stored in a child text node.
            - <**variable**>: Element of a variable of the mathematical expression. Each variable of a mathematical expression must be described by a <variable>-element! Has attributes *name* (a string with the name of the variable) and *placeId* (the ID of the place which represents the variable). Variables *pi* and *Time* need not to be specified.

3.2 Random number generator

The Pseudo-Random Number Generator (PRNG) used by simulation modes is described in [7]. The Java-implementation is taken from `http://www.javamex.com/tutorials/random_numbers/numerical_recipes.shtml`. By default the `System.nanoTime()`-value is used as the seed. Setting the new seed is implemented as a creation of a new Random-object with the given seed.

3.3 Drawing waiting times in stochastic mode

At each simulation step of the stochastic mode a transition with the lowest waiting time is fired. Waiting times are computed according to the firing rates of the transitions. First, a uniformly distributed random number *rand* in the range between 0 and 1 is drawn using the RNG. If a transition T_i has a reaction rate $r(T_i)$, the waiting time $dt(T_i)$ of that transition is

$$dt(T_i) = -\frac{ln(1 - rand)}{r(T_i)}.$$

If the firing rates should be marking dependent, the static firing rate is multiplied by the *enabling degree* q of the transition. The enabling degree is computed as the minimum of the fractions of the token numbers on the pre-places to the edges' weights between the pre-places and the transition. If several transitions have the lowest waiting time, one of them is selected randomly with equal probability.

To speed up the simulation, waiting times of the transitions are only recomputed if the number of tokens on their pre-places has changed in the last firing. Waiting times of the post-transitions of constant places are recomputed in every step.

3.4 Gillespie SSA implementation

The Gillespie SSA mode implements the Stochastic Simulation Algorithm (SSA) of coupled chemical systems which was first described by Gillespie [3]. It describes the development of a system in a physically plausible way by simulating the occurrences of the chemical reactions utilizing the knowledge of the mass action kinetic. It is an alternative to the widely used *deterministic* modeling and simulations, which model chemical reactions by a set of Ordinary Differential Equations (ODE).

3.4.1 Converting input data

In contrast to conventional ODE-based approaches, the SSA operates with the number of molecules and not with the concentrations. To not confuse the user with unconventional data, simulation setup input is expected in concentrations for the initial compound state and reaction rate constants. Additionally, the user must define the volume of the simulated environment. This input data is then converted to the molecule-based stochastic data.

The user can provide the *number of molecules* for a non-constant place by right-clicking on it in the NetViewer and selecting **"Set the number of tokens for this species"**. However, if the same is done for a constant place, all entered

numbers are regarded as concentrations (in mole per liter) and are transformed to molecule numbers by multiplying them by the volume and the Avogadro constant:

$$n_X = N_A \times [X] \times V$$

with n_X is the molecules' number of compound X, $[X]$ is the concentration of compound X, $N_A = 6 \times 10^{23}$ is the Avogadro constant and V is the volume of the environment in liter.

Also, all values which are provided via the **"Enter simulation data"**-dialog are considered as concentrations and are transformed.

The reaction rate constant k is given in $M \times s^{-1}$ for zero order reactions, s^{-1} for first order reactions and $M^{-1} \times s^{-1}$ for second order reactions. This *deterministic* rate constant k must be transformed into a *stochastic* rate constant c. For an x-order reaction and a volume V, the stochastic rate constant is in inverse proportion to V^{x-1} [6]. The volume is multiplied by the Avogadro constant. Furthermore, for y identical molecules involved in the reaction, the stochastic rate constant is proportional to $y!$. The number of identical molecules involved in the reaction is given by the weight of the edge between a pre-place and the transition. With this knowledge, the stochastic reaction rate constant of a reaction represented by transition T_i can be computed as the deterministic rate constant times $\prod_{j \in \bullet T_i} w_j!$ (with w_j is the weight of the edge between pre-place j and transition T_i) divided by $V^{x-1} \times N_A$ (with x is the sum of the weights of the edges between T_i and its pre-places):

$$c = \frac{k \times \prod_{j \in \bullet T_i} w_j!}{V^{x-1} \times N_A}. \tag{1}$$

Because only reactions of order zero, one and two can be simulated by SSA, equation 1 can be simplified to:

$c = k \times V \times N_A$ for a zero-order reaction,

$c = k$ for a first-order reaction,

$c = \frac{k}{V \times N_A}$ for a second-order reaction of the form $A + B \rightarrow C$, and

$c = \frac{2*k}{V \times N_A}$ for a second-order reaction of the form $2A \rightarrow B$.

3.4.2 Performing a simulation step

A single step of the exact simulation algorithm consists of drawing the next firing time and choosing which reaction will occur.

Firstly, the *rates of all active reactions* must be computed. For this, at each step the *deterministic* rate constants of the active transitions are determined

by evaluating the corresponding mathematical expressions. These deterministic rate constants are transformed into the stochastic rate constants c as described above. From the stochastic rate constant $c(T_i)$ of reaction T_i and the current marking the *reaction rate* $r(T_i)$ is computed by multiplying $c(T_i)$ by the number of distinct combinations of involved reactant molecules $h(T_i)$. Let $M = m_1, m_2, m_3, \ldots, m_n$ be a set of educts of reaction T_i (the set of the pre-places of the corresponding transition). Let $|m_i|$ be the number of molecules of the compound $m_i \in M$ which are involved in the reaction (corresponds to the weight of the edge between the educt's place and the transition) and $|M_i|$ the total number of molecules of the compound $m_i \in M$ (the number of tokens on the place of the compound). The number of the distinct combinations of involved reactant molecules is then

$$h(T_i) = \prod_{j=1}^{M} \binom{|M_j|}{|m_j|},$$

and the reaction rate of the reaction T_i is

$$r(T_i) = c(T_i) \times \prod_{j=1}^{M} \binom{|M_j|}{|m_j|}.$$

Secondly, after the rates of all reactions are estimated, their sum is computed. If this sum is zero (which means that no reaction can occur), the simulation is stopped. Otherwise, two uniformly distributed random variables in the range between 0 and 1, U_1 and U_2 are generated. U_1 is used for calculating the next firing time the equation:

$$\text{time of next firing} = -\frac{ln(1 - U_1)}{\text{sum of reaction rates}}.$$

Thirdly, the next occurring reaction is chosen using the random variable U_2. This is done in a while-loop, which iterates over the reactions and sums up their rates. When the sum of the reactions' rates is equal to or greater than the sum of all reactions' rates multiplied by U_2, the algorithm stops the execution of the loop and choses the last processed reaction. In other words, the reaction T_i is chosen to satisfy the following equation:

$$\sum_{j=1}^{i-1} r(T_j) < U_2 \times \text{sum of all rates} \leq \sum_{j=1}^{i} r(T_j).$$

Think of a wheel of fortune where reactions have areas of the size according to their reaction rates.

3.5 Exact SSA of the fast mode

The fast mode was designed to provide high performance simulation of millions of steps in a reasonable time. Amongst other methods, this is achieved by a complete abstraction from the PN structure using some optimizations which will be described here. The algorithm itself is the same one as used in Gillespie SSA (section 3.4).

The PN structure is converted to simpler data structures. The IDs and the names of the places, the marking (the numbers of tokens on the places) and the mathematical expressions of constant places are stored in arrays in identical order. The same is valid for the IDs, the names and the deterministic rate constants of the transitions. Reactions are described as a stoichiometric matrix. Further, for each compound (place) a list of influenced reactions is created. This list stores the indexes of the reactions which have this compound as an educt. For each reaction, a list of its educts and a list of its products are stored, too. Finally, reactions which have at least one educt represented by a constant place are stored in a separate list. This sort of a "dependency graph" allows to limit the number of reactions whose rates must be recomputed at each step.

In the very first step of the simulation, rates of all reactions are computed. In the next steps, only the rates of the reactions whose educt's number was changed in the previous step are recomputed. Also the rates of all reactions which have educts represented by constant places are recomputed, as the number of tokens on a constant place could be changed without being affected by a reaction directly (e.g. time-dependent places).

3.6 Approximate SSA of fast mode

The approximate SSA algorithm is based on the so-called τ*-leaping* method which was originally proposed by Gillespie [4], for the implementation see [5]. In brief, the algorithm chooses a time interval τ and decides how many times each reaction will occur in this period. The time interval should be small enough that the reaction rates do not change significantly during the interval. To avoid negative populations, reactions which can fire maximal 20 times are considered as *critical* and simulated in an exact way only.

At the beginning of each simulation step critical reactions are determined. The algorithm then computes two firing times - the one of the *non-critical reactions* τ_1 and that of the *critical reactions* τ_2. τ_1 is computed according to equation 24 of part 3.2 of the section "Stochastic Simulation for Biochemical Systems" in [5], using the set of non-critical reactions. τ_2 is computed like the waiting time in the exact SSA using the set of critical reactions only.

If $\tau_1 < \tau_2$, no critical reaction will occur in the next step. For each non-

critical reaction, the number of its occurrences is generated as a random Poisson variable with the mean value $r(T_i) \times \tau_1$ and the reaction is executed the chosen number of times. If $\tau_2 < \tau_1$, one critical reaction is chosen like in the exact SSA and the number of occurrences of the non-critical reactions are chosen like in the first case.

4 Simulation of insulin receptor (IR) recycling model - a practical example

This section gives an example of modeling and simulating a biochemical system of insulin receptor (IR) activation and recycling using MonaLisa. It also provides some estimations about the execution speed of the exact and the approximate modes under different conditions. It does not, however, performs an analysis of the IR system as the goal is simply to show the possible workflow scenario. Modeling and simulation of this system is described by Sedaghat *et al.* [8].

4.1 Insulin receptor model

Insulin is an important hormone which regulates various processes, amongst others the glucose intake by the cells. The answer is mediated by the IR which is located in the cell surface membranes. Impairments in the key components of this system may cause such diseases as the *metabolic syndrome* or *Type 2 Diabetes mellitus*.

The PN structure which models the system is depicted in figure 14. The PN consists of 7 places and 11 transitions. The unbound inactive insulin receptor is modeled by the place IR, the free insulin by the place Insulin. We consider that only one cell is simulated and insulin concentration is regulated by external processes, therefore this place is *constant*. Insulin can bind to the free receptor (transition IR_Insulin_Binding). The receptor-ligand complex (place IR_I) can dissociate (transition IR_I_Insulin_Release), and the insulin receptor molecule becomes available for further bindings. Alternatively, the unbound receptor can be internalized to the cytoplasm (transition IR_Inter) and is added to the internal receptor pool (place IR_Int).

As soon as insulin is bound to the receptor, the receptor-ligand complex can be phosphorylated and thus activated (transition IR_I_Phos). The bound phosphorylated receptor is represented by the place IR_I_P. The phosphorylation is reversible (transition IR_I_P_Dephos), the reverse action goes along with the dissociation of insulin. Alternatively, the activated receptor-ligand complex can be internalized (transition IR_I_P_Inter). The phosphorylated complex in cytosol

(place IR_I_P_Int) can be either transported back to the membrane (transition IR_I_P_Int_Deinter) or deactivated in the cell (transition IR_I_P_Int_Dephos). The inactive unbound receptor can then be transported back to the membrane (transition IR_Int_Deinter) or destroyed by degradation (transition Degradation). The population of IR is sustained by the synthesis of the receptor.

Finally, there is a *constant* place IR_Total. This place is not connected with any transition and would violate the standard rules of the PN modeling. However, it can be used in the simulation mode for monitoring the total amount of the insulin receptor (disregarding its state) in the cell.

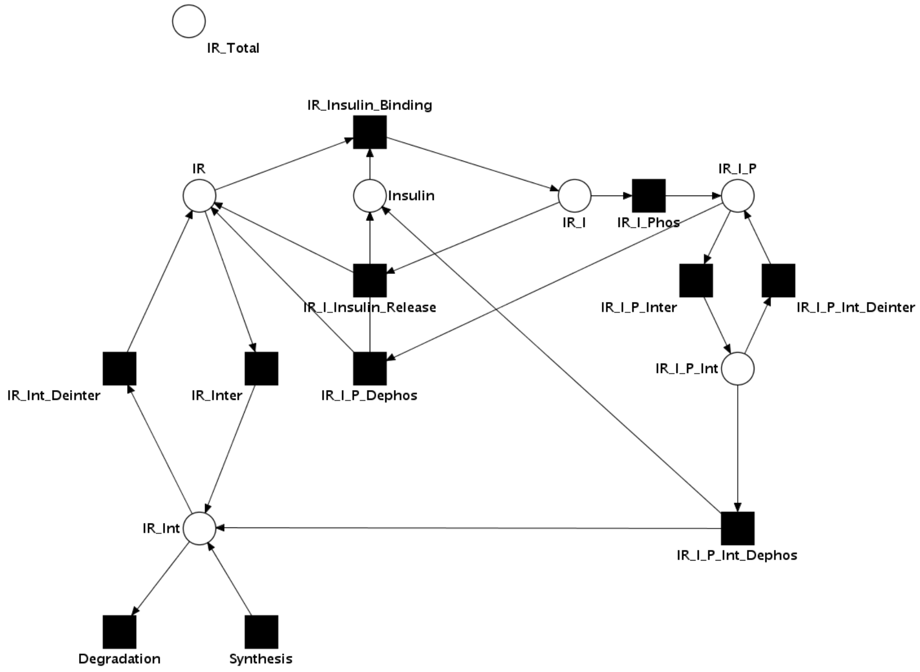

Figure 14: Petri net of the insulin receptor recycling model.

4.2 Simulating the model

We choose the **"Gillespie SSA"**-mode for the simulation of this model. The parameters are adapted from [8]. The volume of the system is set to 1E-9 l. Initial concentration of the membrane located insulin receptor (place IR) is 9E-

13 M, that of the cytosol located receptor (place IR_Int) is 1E-13 M. 1E-9 M of insulin should be applied for 24 hours. The mathematical expression for the place Insulin reads

```
if Time <= 86400 then 1E-9
```

and for the place IR_Total

```
IR + IR_I + IR_Int + IR_I_P_Int + IR_I_P.
```

The (deterministic) reaction rate constants are shown in figure 15. The rate of IR synthesis is dependent on the concentration of the receptor in the cytosol. If the concentration decreases below the steady state value of 1E-13 M, the accelerated synthesis rate is used. This is described by the expression

```
if IR_Int+IR_I_P_Int >= 1E-13 then 2.78E-19;
1.67E-18.
```

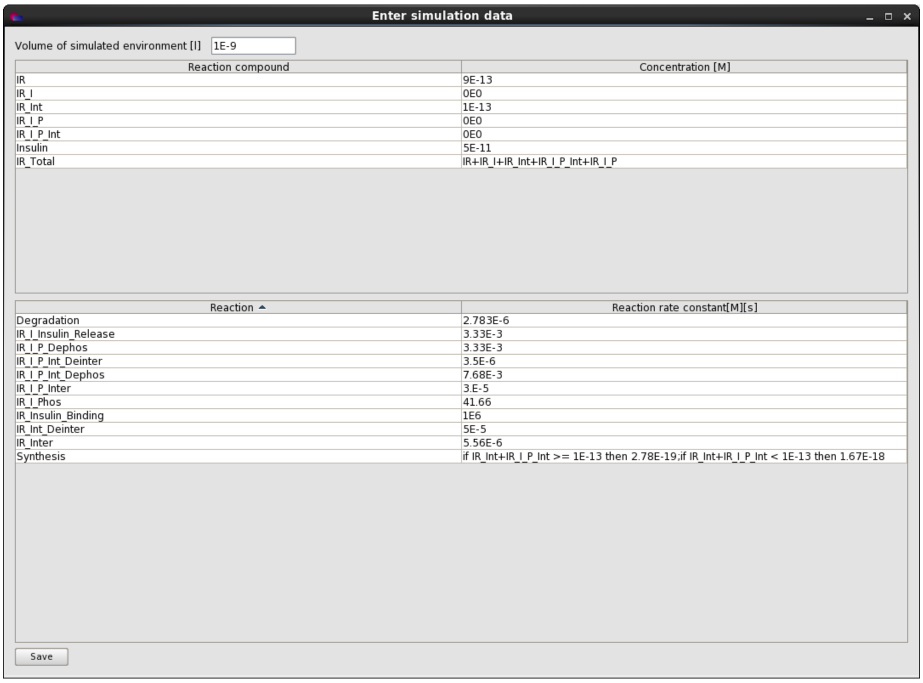

Figure 15: Simulation parameters of the insulin receptor recycling model.

Both, the PN (as a MonaLisa-project) and the simulation setup files, are available to download.

Now, we choose the **Fast simulation mode**. Simulating the system with the exact SSA for two days is done within three seconds. In this time, 102531

steps were simulated. The result's file is 6.3 MB large, the plot of the simulation is shown in figure 16.

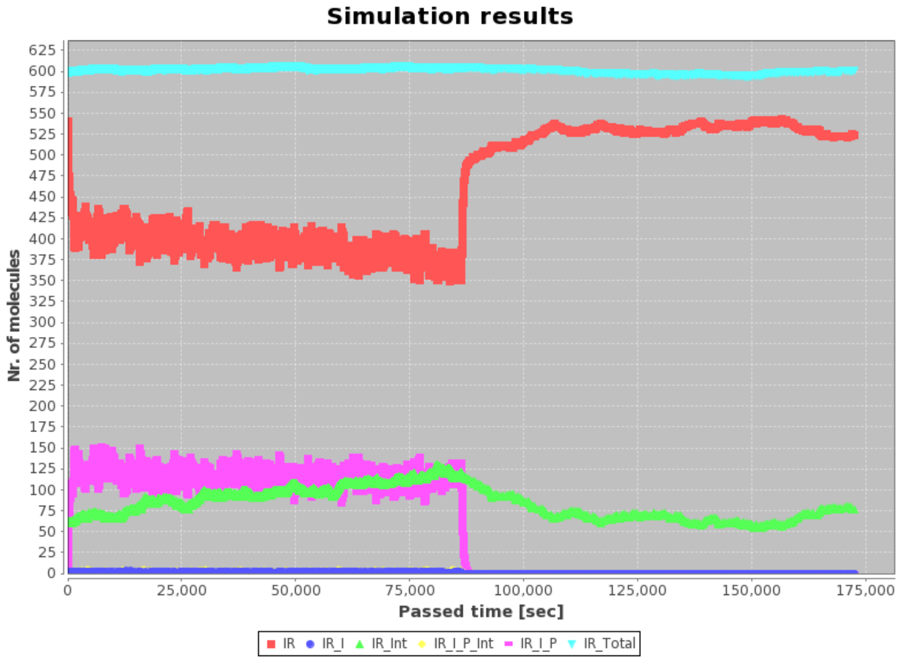

Figure 16: XY plot of the simulation results of IR recycling model.

4.3 Comparing Exact and Approximate SSA

To analyze the performance's differences between the exact and the approximate algorithms, two different simulations were performed with each of the algorithms.

The first simulation was performed on the above described model and parameters. Insulin concentration was set to 1E-7 M, and the system was simulated for 100 days. With this initial settings, 600 insulin receptor molecules are located in the cell, located in the membrane and in the cytosol. The exact algorithm performed 30 407 678 steps in 10 minutes and 5 seconds. The result's file was 2 GB large. The approximate algorithm performed 30 397 468 steps in 11 minutes and 3 seconds, the result's file was also 2 GB large. It is obvious that there is no significant difference between the exact and the approximate algorithms in this simulation.

The second simulation was performed on a slightly different (not published) model of IR recycling. The main difference is that the initial number of receptor molecules in the cell is 330 000. Simulation of the model with insulin concentration of 1E-7 M for one hour using the exact algorithm took 9 minutes and 3

seconds. 22 650 003 steps were simulated and the size of result's file was 2 GB. The same simulation with the approximate algorithm took only 3 minutes and 27 seconds, and the file size was 10.1 MB. The time-plot of the results was comparable to that of the exact algorithm. It is noticeable that stochastic effects are hardly observable with such a huge number of molecules. This setup shows the strength of the approximate algorithm for big systems or high concentrations of the involved compounds. It produces authentic results much faster than the exact algorithm by simulating multiple reactions in a single simulation step.

5 Elements of the GUI

This section lists and explains all elements of the user interface that are provided by the simulation module.

5.1 "Simulation"-tab of the ToolBar

- **"Simulation mode:"**-drop-down menu: Selects the mode by which the system will be simulated. Once a simulation has been started, the mode can not be changed. To change the simulation mode, the running simulation must be stopped. No parallel simulations of the different modes are allowed. Following modes can be selected (for more details, see **"Step-by-step guide"** 1):

  - *Gillespie SSA* (default value): Simulation of the natural behavior of (bio-)chemical systems. Chemical compounds or states are represented by places and reactions by transitions. Each chemical reaction has a reaction rate constant, chemical compounds have initial concentrations. Simulation mimics the development of the system by "performing" reactions, which take place depending on their rate constants and compound concentrations.
  - *Asynchronous*: Simulation mode in which one transition is fired at each step. The transition is randomly selected from the set of active transitions.
  - *Synchronous*: Simulation mode in which multiple active transitions are fired simultaneously at each step. Concurrent transitions are randomly selected.
  - *Stochastic*: Simulation mode in which one transition is fired at each step. The probability of a transition to be selected is given by its firing rate. Firing rates can be dependent on the activation state

of the transitions (transitions, which are activated repeatedly, have higher firing rates).

- **"Start simulation"**-button: Starts the selected simulation mode. Disables the **"Control"**-tab of the ToolBar. Disables net editing. Enables control elements of the simulation mode. Changes the behavior of the mouse in the NetViewer to the simulation mode (selecting an active transition fires it; selecting a place and right-clicking on it enables the token number input).
- **"End simulation"**-button: Stops the current simulation and switches the controls back to the editing mode. Disables the simulation controls.
- **"Steps to perform:"**-text area (default value 1): Specifies the number of steps the simulator will try to perform. It will stop earlier if no active transition exists.
- **"Continuous mode"**-check box: If selected, simulation steps will be performed until no active transitions left or interrupted otherwise. A firing sequence can be interrupted by pressing the **"Stop simulation sequence"**- or the **"End simulation"**-button or closing the NetViewer.
- **"Start simulation sequence"**-button: Begins the execution of a specified number of simulation steps.
- **"Stop simulation sequence"**-button: Terminates the ongoing simulation sequence.
- **"Statistics"**-button: Opens a window with information about the current simulation state, including the number of performed steps, the total number of fired transitions and the numbers of firings per transition.
- **"Show plot"**-button: Opens a window with an XY-plot of simulation results.
- **"Preferences"**-button: Opens a window for adjusting various simulation parameters.
- **"History of performed steps"**-list: List of the 100 last performed simulation steps. Selecting a step will set the system state (marking) corresponding to the step and update the visual output. The step whose state is currently shown is highlighted by red background. Initiating a new firing sequence will erase all following history entries.

- **“Step back”**-button: If possible (not possible if the currently selected step is the first one), performs a simulation step from the history previous to the selected one.
- **“Step forward”**-button: If possible (not possible if the currently selected step is the last one), performs a simulation step from the history following the selected one.
- **“Snapshots”**-list: List of the simulation snapshots. A snapshot stores the last 100 steps of the simulation and is created after every 100 steps by default. It can be deactivated in the “Preferences”-panel.
- **“Custom markings”**-drop-box: List of the markings (numbers of tokens on places) which can be selected. Selecting a marking applies the stored number of the tokens to all non-constant places. Constant places are ignored. By default, each net has an empty marking. Markings are saved with the views (see NetViewer documentation) and are restored upon loading a project.
- **“Save marking”**-button: Saves the current state (numbers of tokens on places) to a custom marking. Each marking must have a unique name. A marking contains only the numbers of tokens on the places, and no mathematical expressions for constant places. Mathematical expressions of the constant places are evaluated and stored as numbers.
- **“Delete marking”**-button: Deletes the marking which is currently selected in the markings drop-box.
- **“Save setup”**-button: Saves the current simulation setup into an XML file. A simulation setup can contain the number of tokens on the places, constant/non-constant states of places, mathematical expressions of the constant places, firing rates and other settings. Exact settings are specified by the currently used simulation mode.
- **“Load setup”**-button: Loads simulation setup from the selected XML file. The content of the setup file depends on the simulation mode which created the file. Setup files can be created and loaded by different simulation modes. In such case, unsupported features are ignored, as well as the places or the transitions not present in the current net.

5.2 “Simulation preferences”-window

- **“Create log file”**-check box (default disabled): If selected, simulation results are written into a tab stop separated textfile which is stored in the

location defined in the text field below. See section 1.9.1 for details on the file structure.

- **"Save snapshots"**-check box (default enabled): If selected, a snapshot of the system state, containing the last 100 steps, is created every 100th step.
- **"Built-in plotting"**-check box (default enabled): If selected, a plot of the simulation results can be created with the **"Show plot"**-button. If disabled, the **"Show plot"**-button is inactive.
- **"Places to plot"**-button: Enables selecting places whose results will appear in the plot.
- **"Delay (ms)"**-text field (default 0): Time in milliseconds between the firing steps. If 0, firing steps are executed with no delay, otherwise the simulator will hold for the defined time before performing the next step.
- **"Update interval (firings)"**-text field (default 1): The interval between updating the visual output in the NetViewer (i.e. number of tokens on places and transition states), given in simulated steps. If 1, visual output is updated after each simulation step. If 0, the NetViewer is only updated when the simulation sequence stops. Disabling visual update or increasing the interval may improve simulation speed.

5.3 NetViewer in simulation mode

- **Token numbers** are drawn inside places (as red dots for token numbers below 5 or as a digits for token numbers above or equal to 5).
- **Black transition**: Transition is inactive and cannot fire.
- **Black transition with green border**: Transition is active and can fire.
- **Black transition with red border**: Inactive transition which fired in the last step.
- **Green transition with red border**: Active transition which fired in the last step.
- **Left clicking** on an active transition fires it.
- **Left clicking** on a place followed by **right clicking**: opens a pop-up menu:

- **"Set the number of tokens for this species"**: Opens a window where the number of tokens for the place can be entered. Selecting **Cancel** or closing the window discards the changes.
- **"Constant token numbers"**-check box: If selected, the place is *constant*, i.e. the number of tokens on this place will not be modified by the simulation.

5.4 Synchronous mode - specific controls

- **"Fire at once (% of active transitions)"** in the preferences menu (default 100): Defines what fraction (in percent) of active transitions will fire in one step. All transitions are treated equally and selected randomly. The value is rounded up.

5.5 Stochastic mode - specific controls

- **"Simulated time"**-text label: shows the simulated time (in arbitrary units). Updated every time when a firing sequence is completed.
- **"Firing rates"** - button: Opens a window where the firing rates of the transitions can be specified. A firing rate can be altered by double-clicking on it and confirming a new value using the "Enter"-key.
- **"Marking dependent firing rates"**-check box in the preferences menu (default disabled): If disabled, the transition's waiting time dt is given by the firing rate $r(T_i)$ of the transition only and is drawn from the probability density function

  $$f(dt) = r(T_i) \times e^{-r(T_i) \times dt}.$$

  If enabled, waiting times are dependent on the activation degrees of the transitions, i.e. the number of possible firings of the affected transition, at current marking. The waiting times are drawn from the probability density function

  $$f(dt) = q(T_i) \times r(T_i) \times e^{-q(T_i) \times r(T_i) \times dt},$$

  with the activation degree $q(T_i)$.
- **"Set RNG seed"**-button: Opens a window for setting the seed of the random number generator (RNG) used for drawing the waiting times. Two simulations started from identical marking, with identical firing rates and identical RNG seed will always produce identical results. Precisely, a new

RNG is generated each time when the "OK"-button of the seed input window is pressed. The number which is shown in the seed input window by its creation is the currently used seed.

5.6 Gillespie SSA - specific controls

- **"Simulated time"**-text label: shows the simulated time (in seconds). Updates every time when a firing sequence is completed.
- **"Enter simulation data"**-button: Opens a window where the user can enter the parameters of the simulated system, including the volume of the system (in liter), the initial concentrations (in M) of the compounds and the rate constants of the reactions (in $M \times s^{-1}$, s^{-1} or $M^{-1} \times s^{-1}$). Reaction rate constants and concentrations of the constant places are defined as mathematical expressions. Scientific notation is allowed.
- **"Fast simulation mode"**-button: Starts the faster implementation of the Stochastic Simulation Algorithm. This mode uses the PN based description of the system but does not update its state during the simulation. It uses the input data such as volume, initial concentrations of compounds, reaction rate constants and the RNG seed of the Gillespie SSA mode. Up to 500 parallel simulations with different RNG seeds are allowed, and the results are written to separate text files. The fast mode can use the *exact* stochastic simulation algorithm or the *approximate* one for simulation speed up without significant accuracy loss.
- **"Set RNG seed"**-button: Opens a window for setting the seed of the random number generator (RNG) used for drawing the waiting times. Two simulations started from identical marking, with identical firing rates and identical RNG seed will always produce identical results. Precisely, a new RNG is generated each time when the "OK"-button of the seed input window is pressed. The number which is shown in the seed input window by its creation is the currently used seed.
- **"Limit number of threads to:"**-check box: If selected, only the specified number of simulation runs of the fast simulation mode can be executed in parallel. If more runs are started, they will be queued and executed as resources become available.

5.7 Fast simulation mode

- **"Output"**-text field: Location and name of the CSV file in which simulation results will be written. If several simulation runs are started, the

index of the run is appended to the defined name.

- **“Start simulation sequence”**-button: Starts the defined number of simulation runs and executes them until the specified time is simulated or the simulation is aborted by the user.
- **“Stop simulation sequence”**-button: Sends a termination request to all simulation runs. When all runs have successfully terminated their work, the button turns back to **“Start simulation sequence”**.
- **“Select algorithm:”**-drop-box: Allows to select which algorithm should be used for the simulation:
  - *Exact SSA* (default): In each simulation step, the time of the next occurrence and the reaction which will occur are computed. Only one reaction per step is simulated.
  - *Approximate SSA*: Estimates how often each reaction will occur in a specific time interval (time intervals selected dynamically). Speeds up the simulation for the large molecule amounts, but can be less precise on low molecule numbers.
- **“Time span to simulate:”**-text field: Defines the time which should be simulated (days:hours:minutes:seconds). The default value is “000:00:00:00”, which means that the simulation will continue until it is interrupted by the user or no reaction can occur.
- **“Write results into file interval:”**-text field: Interval of simulated time at which the results will be written into the file. The default value is “00:00:00:00”, which means that every step is protocoled.
- **“Number of parallel simulations”**-field: Allows to select up to 500 simulation runs, which will be started with the equal data but different seeds of the RNG.
- **“New random”**-button: Every fast mode simulation which is started with the **“Fast simulation mode”**-button has the same seed of the RNG. This produces identical results if the initial parameters are identical and the seed was not altered. Pressing the **“Next random”**-button sets a new, random seed for the RNG of the fast simulation instance.
- **“Apply state to PN”**-button in the run tab: Writes the actual number of compound molecules into the marking of the PN.
- **“Places to plot”**-button: Opens a window where the user can select which places’ values should be plotted.

- **“Show plot”**-button: Opens a window with an XY-plot of the simulation results of the current run. The values are taken from the output file, so the **“Write results into file interval:”**-text field has the influence on which points will appear in the plot.

6 License

MonaLisa is licensed under the Artistic License 2.0 and depends on non-free software. The text of the license can be found at http://www.perlfoundation.org/artistic_license_2_0.

The plotting functionality if provided ty the **JFreeChart** library [1], which is an open-source library covered by the *GNU Lesser General Public License (LGPL) version 2.1*. The source code of **JFreeChart** is supplied within the MonaLisa-package and the license's text can be found at http://www.gnu.org/licenses/old-licenses/lgpl-2.1.html.

7 Frequently Asked Questions

1. **Q**: The simulation is too slow and consumes lots of memory. How can I speed up the process?

   A: Generally, scientific simulations consume much resources. However, you can speed up the process by disabling visual components and following some rules:

   - Try to avoid mathematical expressions where these are not necessary.
   - Try to use as few variables in the mathematical expressions as possible. Check that no unused variables are selected in the **“Used variables”**-list (see 1.10.1).
   - Disable updating the visual output (number of tokens on the places in NetViewer) or increase the update interval in the **“Preferences”**-window (see 1.9) (speeds up simulation significantly).
   - Disable snapshots (reduces memory consumption) (see 1.9).
   - Disable built-in plotting or reduce the number of plotted places in the **“Preferences”**-window (can consume a lot of memory for thousands of simulation steps).

   Of course, disabling features is annoying and you would like to have great performance with full feature set. Unfortunately, these features consume

computational power at various amounts, so you have to decide for yourself which functions to use in order to find the best balance between the functionality and the performance.

8 Changelog

- **04.03.2014**
  - Fixed a bug which appeared when loading a saved marking
  - Several bugfixes.
- **24.02.2014**
  - Number of parallel running threads accords to the number of processors (logical, e.g. multithreaded cores are considered as extra processor) the JVM (Java Virtual Machine) can access. Usually the JVM can utilize al cores of the machine, but it can be limited by user or OS. This step should guarantee that the simulation can utilize all available cores and keep the overhead low.
- **21.02.2014**
  - Performance improvement
  - Factorial calculation fixed. **WARNING!** Any SSA-simulations on nets which have edges with weights ≥ 2 performed with earlier versions of simulator are invalid! This update fixes the wrong behavior. However, the problem only applies to (rare) reactions of the form $2A \rightarrow B$.
- Mathematical expressions have a new function for integer division **div(x,y)**.
- Number of parallel simulation runs increased to 500. The actual number of parallel threads is limited to 50, remaining runs are queued and executed upon finishing previous runs.
- Performance of parallel simulation runs improved.
- Confirmation-dialog when trying to close **Fast simulation mode** with running simulations added.

9 Acknowledgments

Ina Koch, Jörg Ackermann, Klaus Lindauer for supervising. Jens Einloft for support of MonaLisa and cooperation on implementation of simulator. Jennifer

Scheidel and Leonie Amstein for testing. Andreas Blaumeiser for proofreading the documentation.

References

[1] jFreeChart. [Online; accessed 03.24.2014].

[2] Frank Asseg. exp4j. [Online; accessed 03.24.2014].

[3] Daniel T Gillespie. Exact stochastic simulation of coupled chemical reactions. *The Journal of Physical Chemistry*, 81(25):2340–2361, 1977.

[4] Daniel T Gillespie. Approximate accelerated stochastic simulation of chemically reacting systems. *The Journal of Chemical Physics*, 115:1716, 2001.

[5] Lenwood S. Heath and Naren Ramakrishnan. *Problem Solving Handbook in Computational Biology and Bioinformatics*. Springer-Verlag New York, Inc., New York, NY, USA, 1st edition, 2010.

[6] Thomas G. Kurtz. The relationship between stochastic and deterministic models for chemical reactions. *The Journal of Chemical Physics*, 57(7):2976–2978, 1972.

[7] William H Press. *Numerical recipes, 3rd edition: The art of scientific computing*. Cambridge University Press, 2007.

[8] Ahmad R Sedaghat, Arthur Sherman, and Michael J Quon. A mathematical model of metabolic insulin signaling pathways. *American Journal of Physiology-Endocrinology and Metabolism*, 283(5):E1084–E1101, 2002.
